# The germline factor DDX4 contributes to the chemoresistance of small cell lung cancer cells

**DOI:** 10.1038/s42003-023-04444-7

**Published:** 2023-01-18

**Authors:** Christopher Noyes, Shunsuke Kitajima, Fengkai Li, Yusuke Suita, Saradha Miriyala, Shakson Isaac, Nagib Ahsan, Erik Knelson, Amir Vajdi, Tetsuo Tani, Tran C. Thai, Derek Xu, Junko Murai, Nikos Tapinos, Chiaki Takahashi, David A. Barbie, Mamiko Yajima

**Affiliations:** 1 Department of Molecular Biology Cell Biology Biochemistry, Brown University, 185 Meeting Street, BOX-GL277, Providence, RI 02912 USA; 2 Department of Medical Oncology, Dana-Farber Cancer Institute, Boston, MA 02215 USA; 3 Department of Cell Biology, Cancer Institute, Japanese Foundation for Cancer Research, Tokyo, Japan; 4 Division of Oncology and Molecular Biology, Cancer Research Institute, Kanazawa University, Kanazawa, Ishikawa 920-1192 Japan; 5 Laboratory of Cancer Epigenetics and Plasticity, Department of Neurosurgery, Brown University, Providence, RI 02903 USA; 6 Department of Chemistry and Biochemistry, The University of Oklahoma, Norman, OK 73019 USA; 7 Mass Spectrometry, Proteomics and Metabolomics Core Facility, Stephenson Life Sciences Research Center, The University of Oklahoma, Norman, OK 73019 USA; 8 Department of Informatics and Analytics, Dana-Farber Cancer Institute, Boston, MA 02215 USA; 9 Institute for Advanced Biosciences, Keio University, Tsuruoka, Yamagata 997-0052 Japan

**Keywords:** Cancer, Pluripotency

## Abstract

Human cancers often re-express germline factors, yet their mechanistic role in oncogenesis and cancer progression remains unknown. Here we demonstrate that DEAD-box helicase 4 (DDX4), a germline factor and RNA helicase conserved in all multicellular organisms, contributes to increased cell motility and cisplatin-mediated drug resistance in small cell lung cancer (SCLC) cells. Proteomic analysis suggests that DDX4 expression upregulates proteins related to DNA repair and immune/inflammatory response. Consistent with these trends in cell lines, DDX4 depletion compromised in vivo tumor development while its overexpression enhanced tumor growth even after cisplatin treatment in nude mice. Further, the relatively higher DDX4 expression in SCLC patients correlates with decreased survival and shows increased expression of immune/inflammatory response markers. Taken together, we propose that DDX4 increases SCLC cell survival, by increasing the DNA damage and immune response pathways, especially under challenging conditions such as cisplatin treatment.

## Abstract (web summary)

DDX4, a conserved germline factor and RNA helicase, increases small cell lung cancer cell survival by regulating DNA damage and immune response pathways and contributes to cisplatin-mediated drug resistance.

## Introduction

Cancer cells can re-express germline factors typically restricted to gametes^1^. For example, when a malignant brain tumor is induced in *Drosophila* by the inactivation of lethal (3) malignant brain tumor (l(3)mbt) protein, 25% of upregulated genes are germline factors. Further, inhibition of each germline factor (e.g., *vasa, piwi, aubergine*, or *nanos)* halted tumor growth, suggesting tumor dependency^2^. The acquisition of germline characteristics in somatic cells may thus contribute to phenotypes characteristic of stem cells in general, and possibly tumorigenic cells. To date, few mechanistic studies have examined this biological phenomenon in the context of cancer development. This is partly because of the low expression of these germline factors and their complex functions and post-transcriptional modifications that often result in various phenotypes depending on the cell types and developmental context^2–5^. To start addressing this long-standing question in the field, in this study, we focus on one of the germline factors, DDX4, in small cell lung cancer (SCLC).

DDX4 (*Drosophila* Vasa homolog) is one of the most conserved germline factors among all multicellular organisms. It often serves as a metric for germline determination because of its consistent expression across species, yet its function remains poorly understood. It is a member of the DEAD-box RNA helicase family^6–9^, and found to be similar in sequence to eukaryotic initiation factor 4A (eIF4A)^10,11^. It is considered to function as a regulator of mRNA translation in the germline^10–13^. Follow-up reports in *Drosophila* further suggest that Vasa associates with eIF5B, an essential translation initiation factor required for ribosomal subunit joining^14,15^ and is involved in mRNA translation of germline-specific mRNAs in oocytes or germline stem cells^16,17^. Further, more recent reports suggest that Vasa contributes to piRNA biogenesis and transposon silencing in mouse testes^18,19^. A number of these reports over the last several decades suggest that DDX4/Vasa is a conserved germline factor.

Recent reports from our group as well as others, however, suggest that Vasa might also function outside of the germline, such as in embryonic development^20–22^, and in tissue regeneration^23–25^. Vasa expression in these normal somatic cells is tightly regulated and often transiently expressed during a specific step of development. These observations suggest that Vasa may be expressed in the soma only when linked to specific biological events, such as embryogenesis and regeneration, both of which require active cell proliferation and differentiation. On the contrary, in cancer cells, DDX4 appears to be consistently expressed at a low level and plays an essential role in several human cancers, including ovarian cancer^26,27^, multiple myeloma, and leukemia^28^. Based on these observations, we hypothesize consistent DDX4 expression alters protein synthesis both spatially and temporarily, resulting in increased cell survival. However, the physiological significance of DDX4 in oncogenesis and tumor progression remains poorly described.

SCLC is characterized by high rates of metastasis and relapse after treatment, leading to a dismal survival rate^29^. No targeted therapy is approved for SCLC at current, partly due to a lack of molecular targets to explain the strong neoplastic features such as high rates of metastasis and chemoresistance. In this study, we demonstrate that DDX4 expression increases drug resistance, motility, and mRNA translation, which contributes to challenging clinical characteristics of SCLC. These findings provide an initial understanding of how DDX4 contributes to cancer neoplasticity while identifying a potential therapeutic target in cancers that express DDX4 in the future.

## Results and discussion

### DDX4 is enriched on the mitotic apparatus and its overexpression and depletion change cell morphology in SCLC cells

**Fig. 1:**
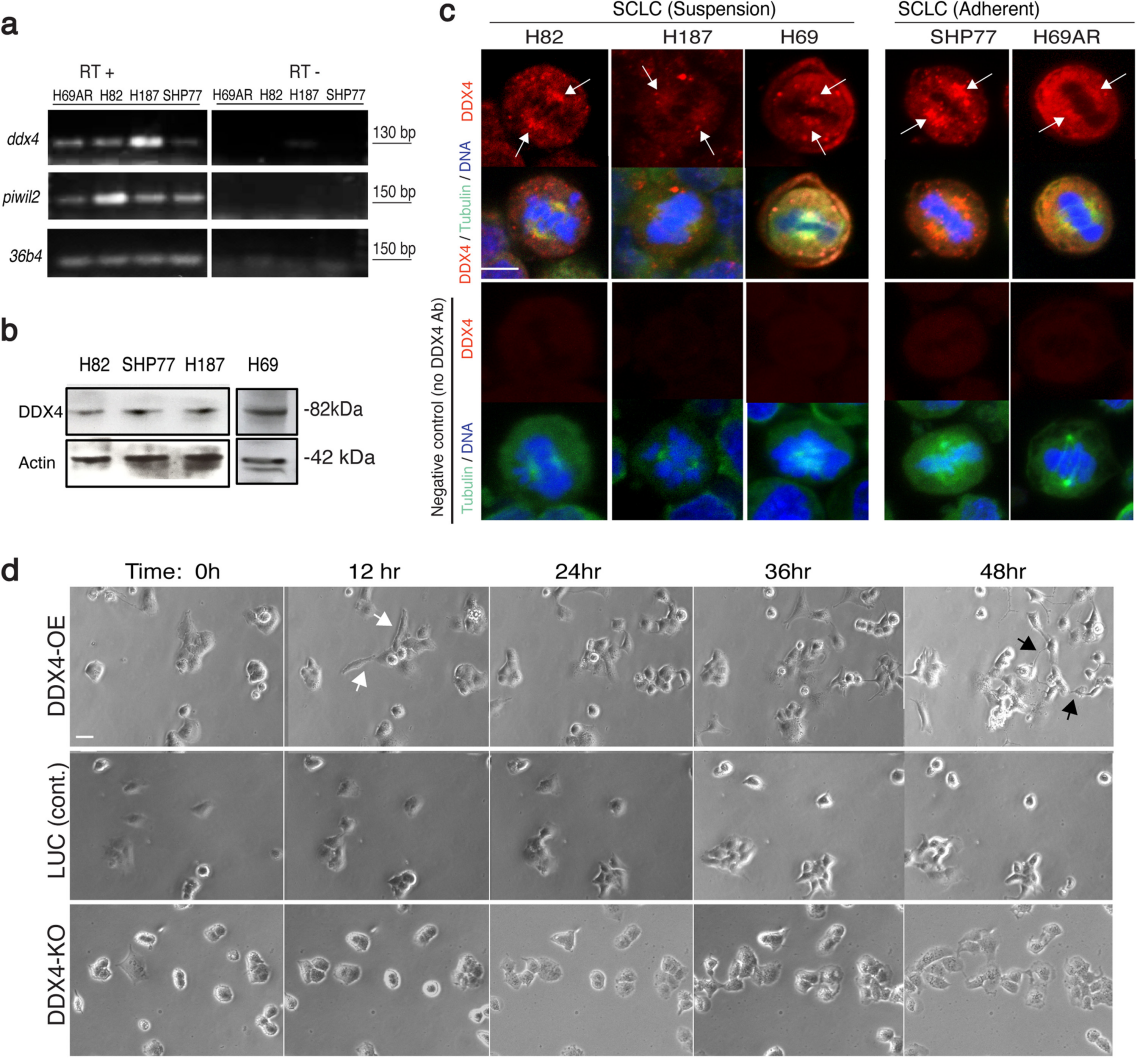
DDX4 is expressed in several SCLC cell lines. **a** RT-PCR results of four SCLC cell lines for three gene products (*ddx4, piwil2, and 36b4*), with negative controls (lacking RT enzyme). **b** DDX4 immunoblot of several SCLC cell lines. Actin was used as a loading standard. **c** DDX4 immunofluorescence results in five SCLC cells counterstained with tubulin and DNA. Arrows indicate the mitotic spindle. Images were taken at a time with the same experimental and laser condition for all cell lines. **d** Time-lapse images of DDX4-OE cell lines show random transformation into the mesenchymal state (white arrows) and increased cellular projections (black arrows), while DDX4-KO cells stayed more rounded. LUC cells are shown as a control. Scale bar = 5 μm.

We previously identified several dozen-cancer cell lines that are potentially expressing *ddx4* transcripts through database searches using the Cancer Cell Line Encyclopedia (CCLE)^30^ and Cancer Genomics Program (CPG, http://www.cancergenomicsprogram.ca/about-cgp)^28^. Among them, 16 lines are blood cancer cells, four of them are ovary/germ cell-derived, and another four of them are SCLCs. Using this database search result, we first profiled *ddx4* mRNA and protein expression in several SCLC cell lines. In the SCLC cell lines tested, *ddx4* and another germline marker *piwi-like2* (*piwil2*) were detected by RT-PCR (Fig. 1a). Since many germline factors, including *ddx4* and *piwi*, are known to undergo extensive post-transcriptional and post-translational regulation^25,31^, mRNA expression may not necessarily reflect their protein expression levels. Therefore, its expression was also confirmed by the immunoblot (Fig. 1b). Immunofluorescence suggests that DDX4 protein is enriched on the mitotic apparatus during M-phase in those cells (Fig. 1c, arrows). Especially in H69AR and SHP77 cells that share a mesenchymal morphology and are partially adherent, DDX4 is stained in the cytoplasm and on the mitotic apparatus. DDX4’s localization to the mitotic apparatus was previously seen in hematopoietic cancer cell lines^25^ as well as sea urchin embryonic cells^20,22^, suggesting DDX4’s possible conserved function among various cells and organisms.

To test the function of DDX4 in SCLC, we used some of those DDX4-expressing cells such as H69AR and SHP77 cell lines and constructed DDX4 overexpression (OE) cell lines that are driven by the EF1α promoter along with controls that are introduced with NanoLuc (Supplementary Fig. S1a, b). EF1α is a strong promoter and is used here to over-emphasize the DDX4 expression phenotypes for the downstream analyses. As expected, DDX4-OE dramatically altered cell morphology with extended filopodia and a flattened shape (Fig. 1d and Supplementary Movies S1 and 2).

Similarly, we also constructed the DDX4-knockout (KO) cell lines (Sg1–3) both in H69AR (Supplementary Fig. S2) and SHP77 cells (Supplementary Fig. S3) using CRISPR-mediated genome editing^28^. The Sg1 and Sg3 lines showed slower cell proliferation compared to the control SC line (Supplementary Fig. S2f), which is consistent with DDX4 depletion in multiple myeloma cells^28^. CRISPR-mediated KO occurs in a random manner, which may completely inhibit protein expression in some cells or may also result in truncated or mutated proteins that are functional in single-cell clones. To reduce this randomness, we further selected every five cells of the H69AR-Sg1 line that showed the most knockout efficiency (Supplementary Fig. S4a). Each of the isolated colonies was evaluated for DDX4-KO efficiency by genomic PCR and sequencing. As a result, the #2 colony group (DDX4-KO# 2) showed 100% mutation efficiency, and 81% of those (*n* = 21) caused a frameshift within or right after the 3rd exon (located between 128 and 205 bp of the total 2073 bp) of DDX4 ORF, significantly reducing DDX4’s expression in the population (Supplementary Fig. S4B, C). Of note, we also attempted single-cell selection with 100% frameshift efficiency within the 3rd exon of DDX4. However, DDX4-KO cells failed to proliferate after single-cell selection whereas control cells grew well under selection, suggesting that some minimal DDX4 expression is critical for cell survival. Although resultant KO efficiency varies among the three pooled clones of DDX4-KO cell lines (H69AR DDX4-Sg1~3, SHP77 DDX4-Sg1~3 and H69AR DDX4-KO# 2 and KO#1 lines), a similar phenotype was observed for all of them: DDX4-KO cells display a rounded cell shape with less adhesive feature compared to controls (Fig. 1d and Supplementary Movies S3 and 4). These results suggest that both the bulk and five-cell selection cell lines demonstrate similar phenotypes caused by DDX4 depletion.

Unlike normal cell lines, cancer cell lines harbor heterogeneity which is known to play an important role in tumorigenesis. This heterogeneity is common even in individual cells within the established cell line^32^. To maintain the minimum heterogeneity of the cell lines, we used these five-cell selection DDX4-KO lines (H69AR DDX4-KO#1–2) as well as bulk DDX4-OE/KO cell lines for further study.

### DDX4 increases cell motility and resistance to cisplatin

**Fig. 2:**
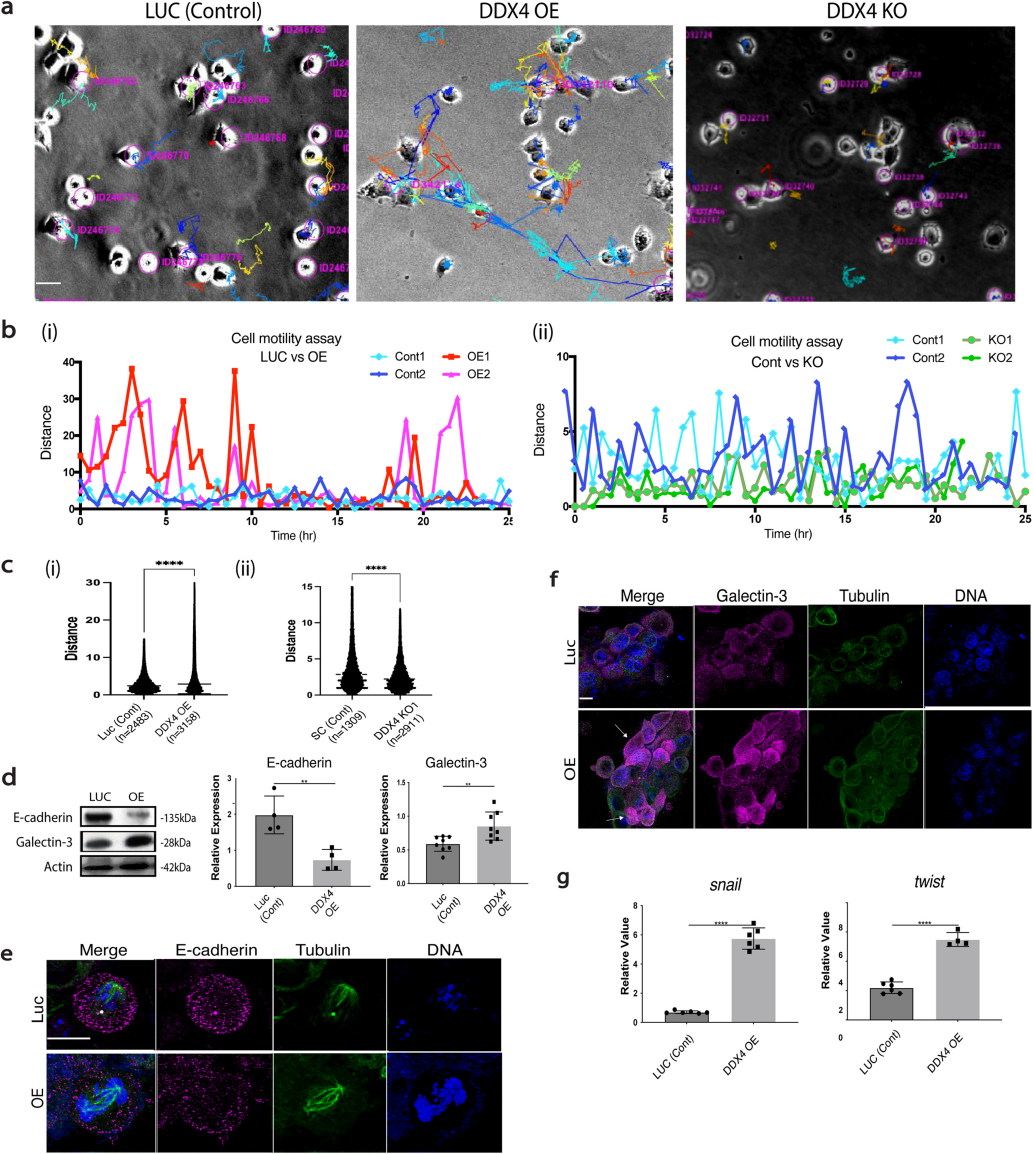
DDX4 expression increased cell motility and metastatic phenotypes. **a**–**c** Cell motility analysis of DDX4-OE and -KO lines in H69AR cells. Each cell line was time-lapse-imaged every 30 min for ~48 h. **a** Each colored line indicates a track of each cellular movement. The longer line suggests the larger movement. **b** Cell motility analysis of a representative cell from each cell line. A distance of travel at each time point of time-lapse imaging was calculated and compared between DDX4-OE and LUC and DDX4-KO and SC. **c** Travel distances per 30 min of all cells in the field were determined by summating and averaging the distance value at each time point for each cell in the field. () indicates the total number of cells analyzed. **d** Immunoblot results of E-cadherin and Galectin-3. The signal intensity of each protein was normalized by that of Actin to obtain the relative value shown in the graph. *n* = 4 biologically independent experiments. **e**, **f** Immunofluorescence of E-cadherin (**e**) and Galectin-3 (**f**) (magenta). Tubulin (green) and DNA (blue) were counterstained. The level of Galectin-3 level was highly heterogenic even within a population of DDX4-OE cells (arrows). **g** RT-qPCR results of *snail* and *twist*. The expression level of each gene was normalized by that of *36b4*, a housekeeping gene. *n* = 3 biologically independent experiments. An unpaired *t* test was used for all graphs. **P < 0.01; ***P < 0.001; ****P < 0.0001. Columns represent means ± SD or SEM. All scale bars = 5 μm.

To test whether DDX4 expression alters cellular motility, each group of H69AR cells was time-lapse imaged. The motility of each cell was automatically tracked by *FIJI*, which shows each cellular movement as an individual line: The longer line suggests more active cellular motility (Fig. 2a). As a result, DDX4-OE and –KO showed increased and decreased cellular motility, respectively (Fig. 2b, c). Similar trends were seen in SHP77 cells (Supplementary Fig. S5). These results suggest that DDX4 increases cellular motility. Of note, we also attempted a conventional cell invasion assay using a transwell, yet DDX4-OE H69AR cells adhered to each other first and formed a tumor in the well, which physically blocked them from penetrating the membrane under the condition we tested. Therefore, DDX4 appears to increase cell motility and cell–cell interaction.

To evaluate whether DDX4 overexpression increases metastatic capacity, we tested expression levels of metastasis and angiogenesis markers in H69AR cells. In DDX4-OE cells, E-cadherin, a negative marker of metastasis, was downregulated, while positive markers such as Galectin-3 protein and the transcripts of *snail* and *twist* were all upregulated (Fig. 2d–g)^33–35^. Taken together, these results suggest that DDX4 is critical for cell motility and potentially contributes to the metastasis of SCLC cells.

**Fig. 3:**
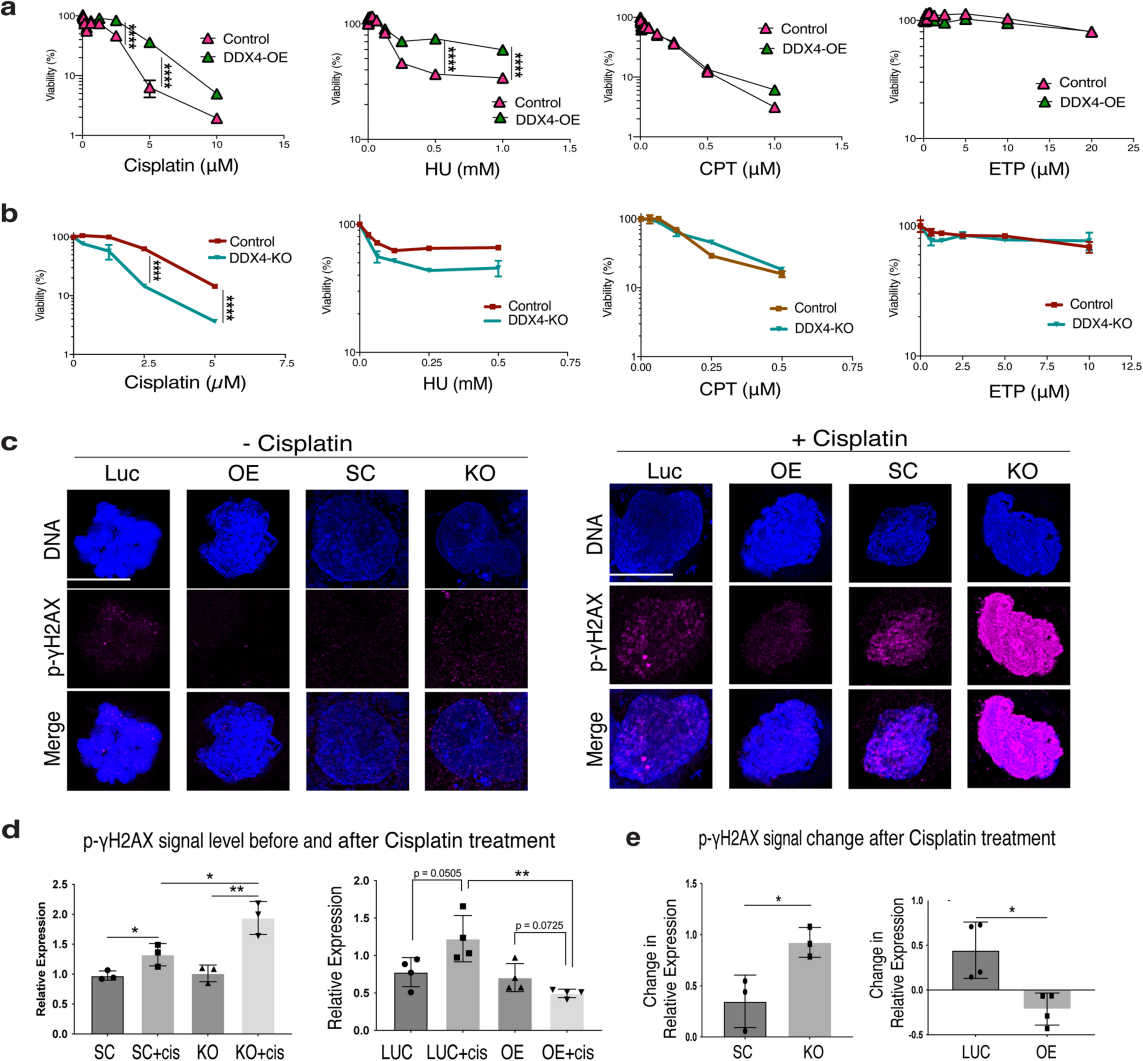
DDX4 promotes resistance to drug treatment and DNA damage. **a**, **b** Cell Viability (CTG) assay results of H69AR DDX4-OE (**a**) and -KO (**b**) cells. Each cell line was treated with various doses of drugs indicated on the X axis for 3 days and the level of viability was measured. Graphs in (**a**, **b**), *n* = 3 biologically independent experiments. **c**–**e** H69AR cells were incubated with the final 10 μM of Cisplatin for 24 hours and then fixed. Phosphorylated γH2AX (magenta) levels were measured using immunofluorescence with levels being lower and higher in DDX4-OE and -KO, respectively. The graphs in (**d**) indicate the quantitative statistical analyses of the phospho-γH2AX signal before and after cisplatin treatment and the graphs in (**e**) indicate the level of signal increase after cisplatin treatment in each cell line. Graphs in (**d**, **e**), *n* = 8 ROIs; *n* = 3 biologically independent experiments. DNA, blue. Scale bar = 5 μm. Error bars shorter than the symbols in (**a**, **b**) are not shown. Two-way ANOVA was used for the graphs in (**a**) and (**b**); unpaired *t* test was used for the graphs in (**d**) and (**e**). **P < 0.01; ***P < 0.001; ****P < 0.0001. Columns represent means ± SD or SEM.

Platinum-based chemotherapy combined with Etoposide is the standard first-line treatment for SCLC, and most patients initially respond. Within a few months, however, SCLC often recurs, leading to devastating outcomes in most patients. To test DDX4’s contribution to cellular survivability, we examined the cell growth and viability of each H69AR cell line under treatment with several anti-cancer drugs including cisplatin, Hydroxide Urea (Hu), Camptothecin (CPT), and Etoposide (ETP). Cisplatin and Hu cause general DNA damage and inhibit DNA repair, respectively, and thus stop cell proliferation. CPT and ETP are both Topoisomerase inhibitors, inhibiting DNA unwinding and chromosome replication. We found that DDX4-OE decreased and DDX4 depletion increased sensitivity to cisplatin and Hu in a dose-dependent manner, while CPT and ETP showed no significant effect in any of the groups tested (Fig. 3a, b). Further, DDX4-OE decreased and DDX4 depletion increased phosphorylated γH2AX (DNA-damage marker) slightly before cisplatin treatment, but more significantly, after cisplatin treatment (Fig. 3c, d). Collectively, these results suggest that DDX4 increases resistance to cisplatin-mediated DNA damage. Since DDX4 is generally considered to be involved in the collective migration of germ cells and maintenance of the genome integrity, it may similarly contribute to SCLC cell metastatic activity and resistance to DNA damage.

### Conserved function of DDX4 on the mitotic apparatus in somatic cells

**Fig. 4:**
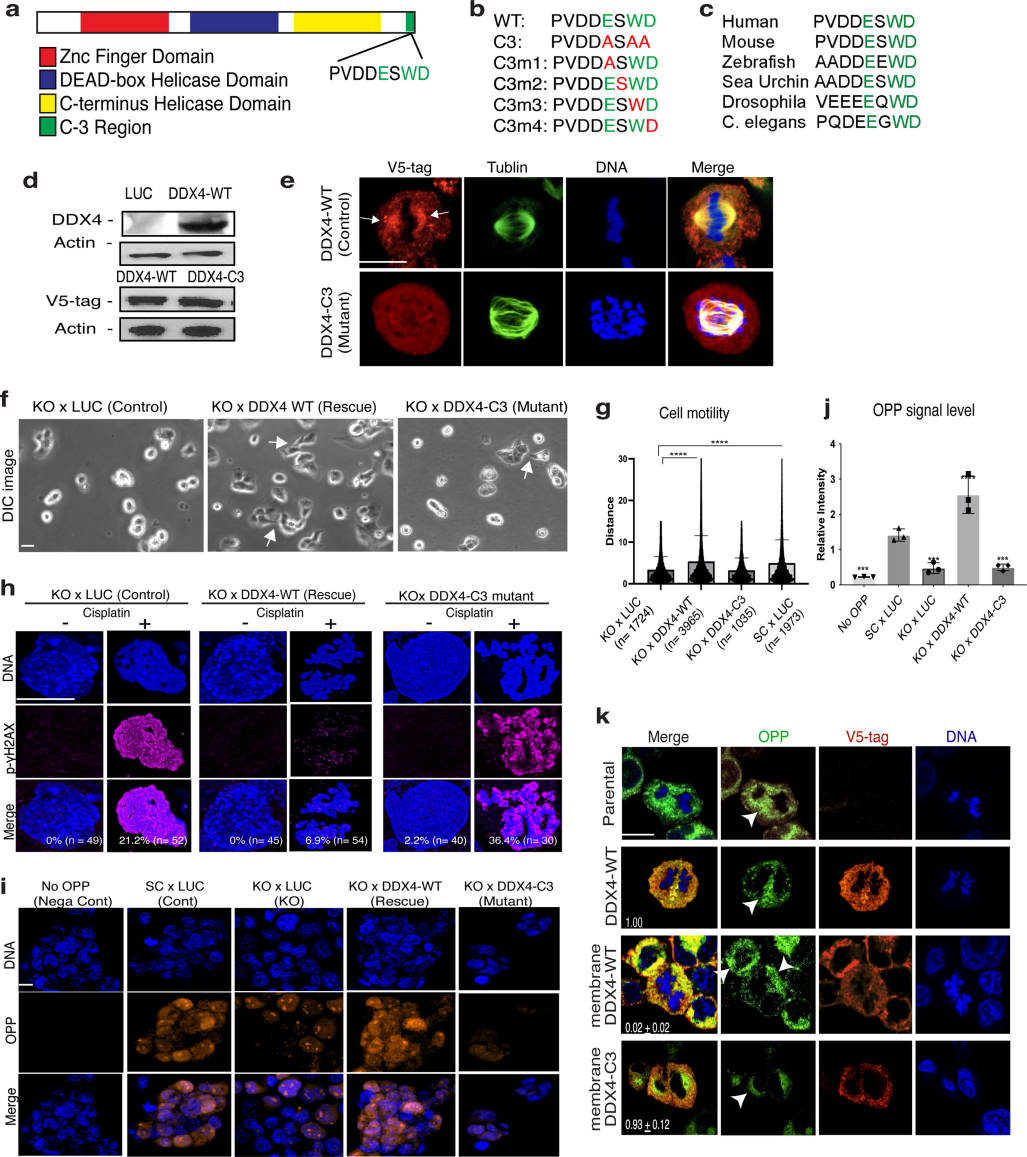
DDX4-C3 region is critical for restoring the DDX4-KO phenotypes in H69AR cells. DDX4-WT or DDX4-C3 mutant was introduced into the DDX4-KO cell line and tested for cell motility and resistance to DNA damage. **a** A schematic diagram showing the DDX4 molecular structure. The C3 region (green) is located at the end of ORF. **b** The amino acid sequence of the DDX4-C3 region is shown. The three amino acids colored in green (E, W, D) in the wild type (WT) were converted to Alanine (A) in the C3 mutant. One each of the three amino acids was converted to Alanine in a single-point mutant (C3m1~4). **c** The sequences of the DDX4/Vasa-C3 region for various organisms are shown. The three amino acids colored green are highly conserved among organisms. **d** Immunoblot results of DDX4 and DDX4-C3. Upper panel, DDX4 antibody was used to detect DDX4. Lower panel, a V5-tag antibody was used to detect DDX4-WT or DDX4-C3 to exclude the endogenous level of DDX4. Actin was used as a loading standard. **e** Immunofluorescence results of DDX4-WT and DDX4-C3 (red, detected by V5-tag antibody). Signal enrichment on the spindle (arrows) found in the DDX4-WT cell was little found in the DDX4-C3 cell. Tubulin, green; DNA, blue. Scale bar = 5 μm. **f**, **g** DDX4-WT rescued the phenotype with extended lamellipodia (**f**, arrows) and cell motility (**g**), while the DDX4-C3 mutant did not. () indicates the number of cells analyzed. **h** Each cell line was incubated with the final 10 μM of Cisplatin for 24 hours and then fixed for phospho-γH2AX (magenta) immunofluorescence. DNA, blue. The percentage in the corner of each image indicates the proportion of the cells showing the positive p-γH2AX signal in each ROI. *n* indicates the total cell number analyzed in the eight ROIs. Of note, the signal level was in general higher in the control (LUC) and the C3 groups. **i**, **j** A level of mRNA translation was detected by OPP (orange). The signal intensity of OPP was normalized by that of DNA (blue) for each sample to obtain the relative signal level shown in (**j**). The values are the average of the eight ROIs through the three replicates. **k** Detection of translation (green, detected by OPP) in H69AR human cancer cell line introduced with DDX4 wild type (WT), membrane-targeted DDX4-WT, or membrane-targeted DDX4-C3 mutant (orange, detected by V5-tag antibody). The OPP signal was found enriched along the chromosomes or in the cytoplasm in the control (Parental and DDX4-WT) and membrane DDX4-C3 mutant groups but was found recruited to the membrane region in the membrane DDX4-WT group (arrowheads). Representative phenotypes are shown (*n* = 10 ROIs). The number in each image indicates the average relative survivability of each cell line against the control line (DDX4-WT; set as 1) at 2 weeks after antibiotic selection (*n* = 3). An unpaired *t* test was used for the graph in (**g**); one-way ANOVA was used for the graph in (**j**). **P < 0.01; ***P < 0.001; ****P < 0.0001. Columns represent means ± SD or SEM. Scale bars = 5 μm.

To further support the above findings, we performed the rescue experiments using the wild-type (WT) DDX4 and the C-terminus mutant (C3). This DDX4-C3 mutant contains three amino acid mutations in the C-terminus (Fig. 4a, b). This region is evolutionarily conserved yet distinct from other DDX helicases (Fig. 4c)^9^, and was also recently reported to be critical for Vasa’s function in embryonic cells of the sea urchin^22^. We, therefore, hypothesize the C3 region is critical for DDX4’s activity in somatic cells.

Both the DDX4-WT rescue line and the DDX4-C3 mutant line showed the same level of expression (Fig. 4d) yet its localization on the spindle and the granular appearance was lost in the DDX4-C3 mutant cell line (Fig. 4e). Recent reports suggest that DDX4 appears as granules by undergoing phase separation^36,37^, the C3 region may be critical for controlling its condensation and localization on the spindle. In those cell lines, we found that DDX4-WT rescued the mesenchymal morphology (Fig. 4f, arrows) as well as cell motility (Fig. 4g), while DDX4-C3 failed to rescue these features of H69AR cells. Consistently, DDX4-WT also rescued chemoresistance, while DDX4-C3 failed in H69AR cells (Fig. 4h).

Further, DDX4 is an RNA helicase and may contribute to these phenotypes by rapidly changing protein expressions in the cells. To test this hypothesis, the general mRNA translation of each cell line was analyzed by the signal level of O-propargyl-puromycin (OPP) that is incorporated into the active translational machinery and thus serves as a translation marker. DDX4-WT rescued translation, increasing it by 100% over the control, while DDX4-C3 showed a ~70% decrease in translational activity similar to the negative controls (Fig. 4i, j). We also performed these rescue experiments using a DDX4 mutant with a single-point mutation in the C3 region (Fig. 4b, C3m1~m4). Those single-point mutants, however, equally rescued both mRNA translation and DNA-damage resistance capability to some extent (Supplementary Fig. S6), suggesting that the three amino mutation is critical for DDX4’s activities in SCLC cell regulation.

Lastly, to test if DDX4’s localization on the spindle is important for its function in SCLC cells, we introduced membrane-targeted DDX4 (membrane DDX4). Membrane DDX4 caused ectopic translation at the membrane, and cells died within one-two weeks after the construction of this cell line, even though endogenous DDX4 is present in those cells (Fig. 4k, OPP). The rest of the control cell lines including the membrane-targeted DDX4-C3 cells showed no aberrant translation nor defect in cell proliferation. These results support the idea that not the amount but the localization of DDX4 is critical for the survival of SCLC cells. Further, the evolutionary conserved C3-region appears to be the critical domain for this functionality of DDX4, suggesting a possible conserved mechanism of the DDX4 function across cancer and embryonic cells.

The above observations suggest the C3 region of DDX4 is critical for multiple cellular phenotypes such as cell motility and drug resistance in SCLC cells. Although the detailed mechanism of DDX4 function through this C3 region is yet to be identified, in this study, the C3 mutant showed less granule formation and failed to localized on the spindle. This may have impacted the fundamental function of DDX4 by changing its conformation and interactions with other molecules on the spindle, resulting in the failed rescue phenotypes. Another hypothesis is that this C3 region is critical for DDX4’s interaction with translation factors and/or mRNA targets to facilitate the translation of mRNAs directly responsible for the cellular phenotypes. Further, a study in the *Drosophila* germline also suggests that this C3 region is important for its function in piRNA biogenesis^38^. We were unable to test this hypothesis in SCLC cells for this study. However, PIWI, a major factor of piRNA biogenesis appears to be also expressed in SCLC cells (Fig. 1a). Therefore, it is important to test if/how DDX4 regulates piRNA and/or small RNA biogenesis in SCLC cells, contributing to the cellular phenotypes in the future. Further investigation into these mechanistic details is awaited to fully understand the nature of DDX4 function in SCLC cells.

### DDX4 increases DNA-damage response in SCLC cells

**Fig. 5:**
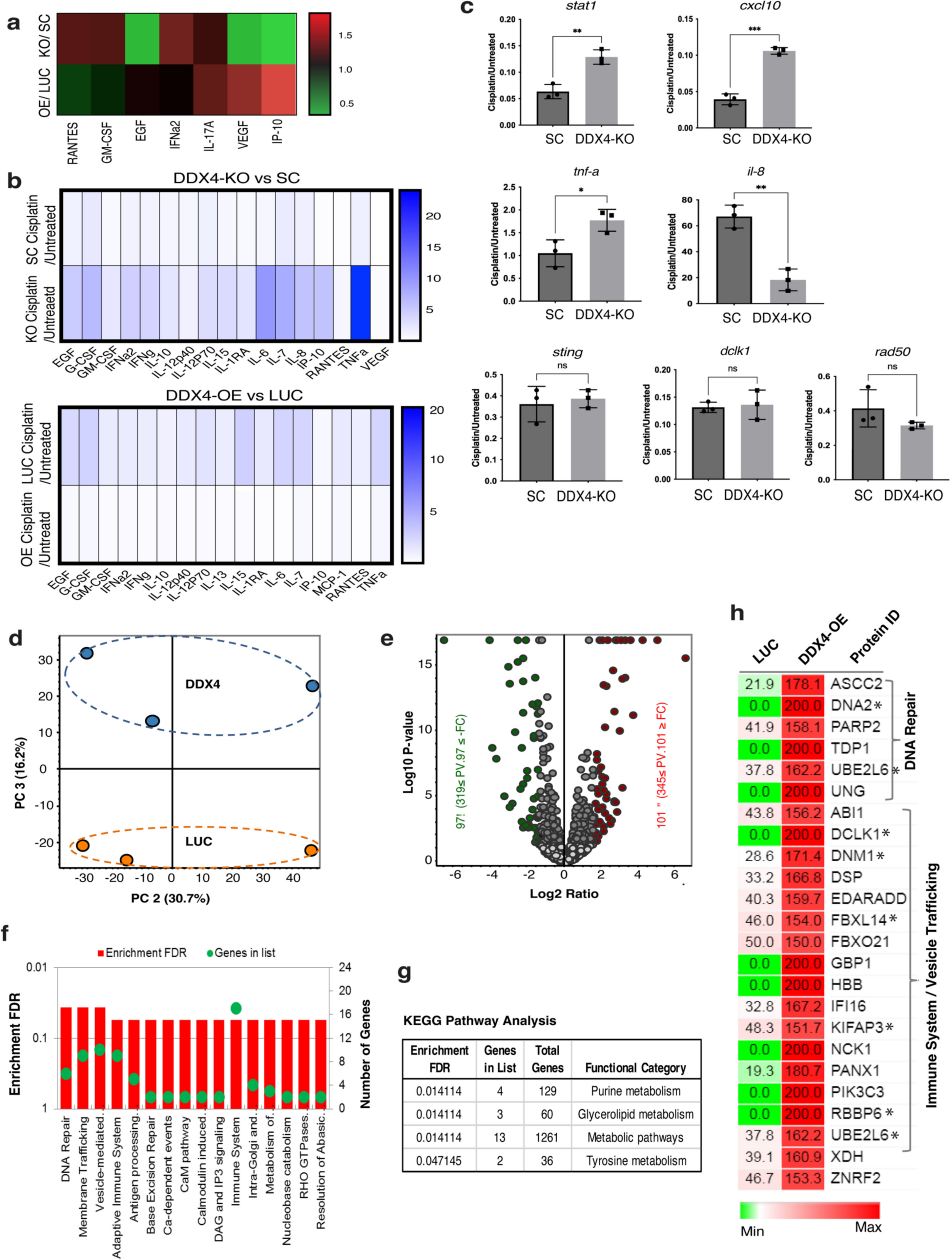
DDX4 globally changes protein expression in H69AR cells. **a** Multiplex cytokine profiling shows DDX4-OE has increased cytokine expression compared to the DDX4-KO cell line at a natural state in H69AR cells. Each value was normalized to that of the corresponding control line for each cytokine. **b** Multiplex cytokine profiling was performed before and after Cisplatin treatment (10 µM Cisplatin treatment for 20 h) for each cell line of H69AR cells. Each value of the “after” treatment group was normalized to that of the “before” treatment group to obtain the relative expression level of each cytokine after Cisplatin treatment. **c** RT-qPCR results of cytokine-related genes. The expression level of each gene was normalized by that of *36b4*, a housekeeping gene to obtain a relative expression level. The graphs are the average of three replicates. **P < 0.01; ***P < 0.001; ****P < 0.0001. Columns represent means ± SD or SEM. **d**–**g** Comparative label-free quantitative proteomic profiling of the DDX4-OE and control (LUC) cell lines. **d** Principal component analysis (PCA) of total protein abundance data collected from DDX4 and LUC samples. Data represents the close clustering of protein abundance of each replicate under the same group, however, showed variability between DDX4 and LUC samples. **e** Volcano plot of fold change versus *q*-value of the total of 4740 proteins quantified from DDX4 and LUC cell lines. Red and green circles represent the significant (*q* < 0.05) up- and downregulated proteins. Gray circles (*q* = 0.05) are nonsignificant and below the threshold of fold expression. Reactome (**f**) and KEGG (**g**) pathway analyses of the significantly increased (red bars) proteins in DDX4-OE vs LUC. **h** Heatmap analysis of the DNA repair and immune system-associated proteins that were significantly increased in abundance in DDX4-OE cell lines. Expression of the proteins with asterisk (*) was validated by immunoblot and/or by immunofluorescence.

Since DDX4 appears to play a major role in mRNA translation, this may allow cells to respond to external cues more effectively and increase cell survival. To test this hypothesis, we analyzed cytokine expression before and after cisplatin treatment in H69AR cells. The multiplex analysis suggests that the cytokine expression profile is markedly different between the DDX4-OE and -depleted groups at baseline (Fig. 5a). After cisplatin treatment, in the group depleted with DDX4, the level of cytokine secretion was globally increased after treatment compared to the control group (SC) (Fig. 5b). RT-qPCR for cytokines and immune signaling components including *stat1* and *cxcl10* also showed an upregulation in the DDX4-depleted group following cisplatin treatment, while the pro-survival factor *il-8* was downregulated after the treatment (Fig. 5c). On the contrary, upregulation of cytokine expression was globally repressed in the DDX4-OE group compared to the control (LUC) (Fig. 5b), making cells insensitive to cisplatin treatment. This may help cells evade immune surveillance and survive during chemotherapy, which needs to be tested in the future using the in vivo system^39–41^.

**Fig. 6:**
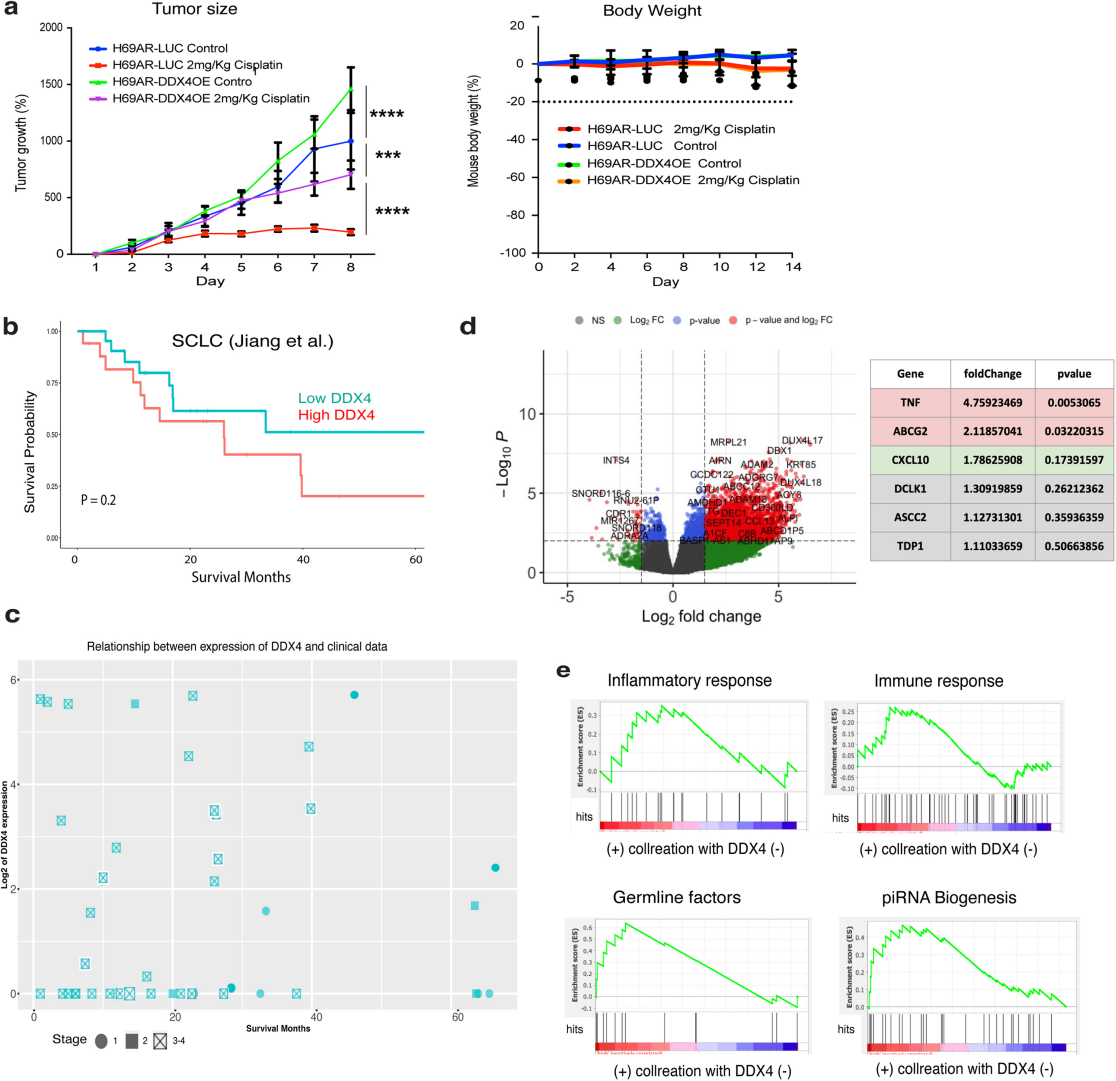
In vivo and clinical implication of DDX4 expression in SCLC and other cancer types. **a** LUC (control) or DDX4-OE H69AR cells were injected into five nude mice in the presence/absence of cisplatin treatment. Tumor size and body weight were monitored over time. Two-way ANOVA was used for the graphs. Error bars mean ± SD or SEM. ***P < 0.001; ****P < 0.0001. **b** Survival analysis of SCLC patient dataset. mRNA expression data of the early stage SCLC was obtained from the previously published study of Chinese patients who were diagnosed with SCLC^42^. The analysis was limited to pre-treatment samples in case chemotherapy alters DDX4 expression. **c** PCA analysis of 40 SCLC samples that are treatment naive and associated with diagnosis using the dataset available in ref. ^42^. A trend of longer survival for SCLC patients was found with low DDX4 expression (log2 (DDX4) < 2) compared with high DDX4 expression (*P* value 0.2). The higher DDX4 expression was also seen mostly in the patients with the more progressed stage (stages 3–4) of cancers. **d** Volcano plot of fold change versus *P* value of the total of genes quantified from DDX4-High and DDX4-Low patients, respectively. Red, green, and blue circles represent the significant (*P* < 0.01; fold change >1.5) up- and downregulated genes. Gray circles (*P* > 0.01; fold change <1.5) are nonsignificant and below the threshold of fold expression. **e** Enrichment analyses of genes for each category. The enrichment score indicates the degree of overrepresentation of each gene set. Red indicates higher rank in the gene set is enriched and blue indicates that a lower rank in the gene set is enriched.

We next performed label-free quantitative proteomics for DDX4-OE and control (LUC) groups in H69AR cells, which identified over 5000 proteins in each group (Fig. 5d–h and Supplementary Data 1). Gene Ontology (GO) analysis indicates that several pathways were upregulated by DDX4-OE, including DNA-damage response, protein/macromolecules modifications, and immune response (Fig. 5f). KEGG pathway analysis suggests that DDX4 alters the metabolism in the cell (Fig. 6g). These proteomics results were further validated by immunoblot and immunofluorescence for representative proteins using both DDX4-OE and -KO cell lines (Fig. 5h and Supplementary Fig. S7a, b). Further, DDX4-OE cells treated with inhibitors of DNA-damage sensing and immune signaling pathways compromised cellular motility (Supplementary Fig. S7c). Taking together, DDX4 appears to increase cell survival by increasing DNA and immune responses, especially under a challenging environment such as the cisplatin treatment in SCLC cells.

To test this hypothesis in vivo, DDX4-OE or control (LUC) H69AR cells were subcutaneously injected into the nude mice, in the presence or absence of cisplatin, respectively. In the absence of cisplatin, the DDX4-OE tumors showed a similar size and weight compared to control (LUC) tumors (Fig. 6a). In the presence of cisplatin, tumor growth was dramatically reduced in the control (LUC) tumors, but not in the DDX4-OE tumors. The body weight of mice was similar among all groups tested, suggesting that DDX4-OE specifically increased drug resistance and promoted tumorigenesis in vivo (Fig. 6a). These results support the contention that DDX4 expression facilitates tumor progression even under a challenging environment such as cisplatin treatment.

### DDX4 expression compromises the survivability of SCLC patients

To assess the clinical implications of DDX4 expression in SCLC, we analyzed publicly available RNA sequencing results of SCLC patients’ samples. mRNA expression data of the early stage SCLC^42^ showed a trend toward worse survival with high DDX4 expression especially at the later stage (Fig. 6b). This suggests that pre-treatment SCLC tumors with high DDX4 expression may be linked to shorter survival in the later stages, while SCLC patients with low DDX4 expression may result in longer survival (Fig. 6b). Another analysis on 40 samples that were treatment naive and associated with the survival data shows a trend of longer survival for SCLC patients with low DDX4 expression compared to the patients with high DDX4 expression (*P* value 0.2) (Fig. 6c), suggests a possibility that DDX4 expression is linked to the survival of SCLC patients, especially toward the late stage. Further, to investigate the potential role of DDX4 in SCLC patients, we performed differential gene expression (DGE) analyses using the same dataset (Fig. 6d). Enrichment analyses suggest that genes involved in immune/inflammatory response were found enriched in the DDX4-high patient groups (Fig. 6d, e and Supplementary Fig. S8), which is consistent with the proteomics results of SCLC cell lines.

Further, genes involved in germline/piRNA biogenesis were also found enriched in the DDX4-high patient groups (Fig. 6e and Supplementary Fig. S8). This result suggests that a cluster of germline factors is indeed expressed in patient tumors as proposed previously^1^ and their expressions negatively correlate with SCLC patient survivability. What upstream mechanism facilitates DDX4 and other germline factor expressions in somatic cancers is currently unknown and requires further investigation in the near future.

### Implications of DDX4 expressions in somatic cancers

To obtain further insight into the clinical implication of DDX4 expression in somatic cancers, we performed the survival analyses using publicly available patient datasets of acute myeloma (AML), esophageal carcinoma (ESCA), lung adenocarcinoma (LUAD), bladder carcinoma (BLCA), and uterine carcinoma (UCA) from TCGA or TARGET (Supplementary Fig. S9). Among those, DDX4 expression compromised patients’ survivability in AML and ESCA, while it showed no significance or the opposite trend in LUAD, BLCA, and UCA (Supplementary Fig. S9). These results suggest that DDX4 expression could contribute to tumorigenesis in both directions. Importantly, the maximum expression range of DDX4 in SCLC, AML, and ESCA datasets is above 0.06, while that in other cancers is nearly 0. This implies that DDX4 expression facilitates tumorigenesis only in certain cell types that are equipped to tolerate its higher expression, while other cell types may not tolerate DDX4 expression and die immediately. Indeed, DDX4 expression in somatic cells is usually low and tightly regulated under normal circumstances, and its ectopic expression results in cell death, suggesting its strong potency in somatic cells^22^. This hypothesis though needs to be further tested in the future using multiple cancer cell lines that have various levels of DDX4 expressions.

### Conclusions: DDX4 functions in somatic cancers

DDX4 had been long considered an exclusive germline marker^6–13^, yet we report in this study the essential functions of DDX4 in somatic cancer cell regulation. Since DDX4 expression in cancer cells is low compared to other oncogenic drivers, its potent function in SCLC cells was initially surprising. However, DDX4 is heavily regulated by post-transcriptional and post-translational modifications, and a low transcript or protein level may not directly imply low DDX4 function, which appears to be indeed the case during brain tumor induction in *Drosophila*^2^. Further, past reports in epithelial ovarian cancer patient tissues also suggests that DDX4 expression is rare and consistently found only in a small fraction of tumorigenic cells with a stem cell marker CD133^27^. Therefore, DDX4 expression may be restricted to a small fraction of the tumor or only during specific timing of tumorigenesis. Further mechanistic studies and investigation on DDX4 expressions in various cancer patients’ tissues will be essential to determine its detailed clinical implications and functional mechanisms.

Lastly, since DDX4 is generally not expressed in adult somatic cells^25^, it may serve as a further tumor/testis antigen and/or represent a potential therapeutic target in cancers expressing DDX4. Although germ cells may be also affected, the importance of these side effects will vary depending on the patient’s life stage. The DDX4-C3 mutant which we identified as critical for DDX4’s function in SCLC cells is highly specific to DDX4 but not conserved in other DEAD-box family proteins. Therefore, this region has the potential to serve as a further therapeutic target in the future. Further investigations in these directions will be useful to evaluate DDX4’s potential as a future therapeutic target.

## Methods

### Key resources table

A list of vectors, primers, and gRNA sequences was used in this study.

**Table:** 

Reagent or resource	Source	Identifier
Lentiviral vectors		
plx304/307-DDX4	DDX4 ORF inserted into the vector	PubMed Gene ID 54514
plx304/307-NanoLuc	NanoLuc ORF inserted into the vector	Kitajima et al.^43^
LentiCRISPRv2-DDX4	DDX4 SgRNA sequence inserted into the vector	Schudrowitz et al.^28^
DDX4 gRNA sequences		
SgRNA1	GTTTCCAAAATTCCGCCCAG	Schudrowitz et al.^28^
SgRNA2	TTTGCCTCTGGGCGGAATTT	Schudrowitz et al.^28^
SgRNA3	ATGAAATGATCTCTTCGAGA	Schudrowitz et al.^28^
Scramble SgRNA	ACGCAATCCGCATCCTTACT	Kitajima et al.^43^
Primers for DDX4 mutant construction		
Universal forward primer	GGGACAAGTTTGTACAAAAAAGCAGGCTACCATGGGAGATGAAGATTGGGAAGCAG	N/A
Reverse primer: DDX4-C3	GGGGACCACTTTGTACAAGAAAGCTGGGTGtgCCgcTGAtgCATCATCTACTG	N/A
Reverse primer: DDX4-C3m1	GGGGACCACTTTGTACAAGAAAGCTGGGTGATCCCATGAagCATCATCTACTGGATTG	N/A
Reverse primer: DDX4-C3m2	GGGGACCACTTTGTACAAGAAAGCTGGGTGATCCCATGcCTCATCATCTACTGGATTG	N/A
Reverse primer: DDX4-C3m3	GGGGACCACTTTGTACAAGAAAGCTGGGTGATCCgcTGACTCATCATCTACTGGATTG	N/A
Reverse primer: DDX4-C3m4	GGGGACCACTTTGTACAAGAAAGCTGGGTGAgCCCATGACTCATCATCTACTGGATTG	N/A
Primers for RT-qPCR		
* ddx4*	F: GGAACAGCGCCAAACCCTTAT	Primer Bank ID: 262231851c3
	R: TCTGCTGAACATCTCTACATGCT	
* piwil2*	F: C AGGCAGAGGCCATGTATTTGG	Primer Bank ID: 209180417c1
	R: AAGCATTTCCCGTTTCAGAGG	
* gapdh*	F: GGAGCGAGATCCCTCCAAAAT	Primer Bank ID: 378404907c1
	R: GGCTGTTGTCATACTTCTCATGG	
* 36b4*	F: CAGATTGGCTACCCAACTGTT	NCBI: NM_007475
	R: GGAAGGTGTAATCCGTCTCCAC	
* snail*	F: TGTGACAAGGAATATGTGAGCC	Primer Bank ID: 324072669c2
	R: TGAGCCCTCAGATTTGACCTG	
* twist*	F: GTCCGCAGTCTTACGAGGAG	Primer Bank ID: 4507741a1
	R: GCTTGAGGGTCTGAATCTTGCT	
* stat1*	F: ATCAGGCTCAGTCGGGGAATA	Primer Bank ID: 189458859c2
	R: TGGTCTCGTGTTCTCTGTTCT	
* il-8*	F: TTTTGCCAAGGAGTGCTAAAGA	Primer Bank ID: 10834978a1
	R: AACCCTCTGCACCCAGTTTTC	
* cxcl10*	F: GTGGCATTCAAGGAGTACCTC	Primer Bank ID: 323422857c1
	R: TGATGGCCTTCGATTCTGGATT	
* dclk1*	F: GCTGATTTGACCCGAACTCTG	Primer Bank ID: 4758128a2
	R: AGCCACATACATAACTCTCTCCT	
TNF-α	F: CCTCTCTCTAATCAGCCCTCTG	Primer Bank ID: 25952110c1
	R: GAGGACCTGGGAGTAGATGAG	
* rad50*	F: TGAAAGTGGTGACGAAAGGAAG	Primer Bank ID: 142370362c1
	R: AATGCAGACACATAAGAAGGGAG	
* sting*	F: CACTTGGATGCTTGCCCTC	Primer Bank ID: 296010926c2
	R: GCCACGTTGAAATTCCCTTTTT	

A list of antibodies used in this study.

**Table:** 

Antibody name	Isotype	Cat #	Vendor name	Dilution (used in IB/IF/IHC)
Monoclonal antibodies				
DDX4	Rabbit	8761	Cell Signaling Technology	1:1000 (IB)
CORTACTIN	Rabbit	3503	Cell Signaling Technology	1:1000 (IB)
RAD50	Rabbit	3427	Cell Signaling Technology	1:1000 (IB)
ABCG2	Rabbit	42078	Cell Signaling Technology	1:1000 (IB)
DCLK1	Rabbit	62257	Cell Signaling Technology	1:1000 (IB); 1:200 (IF)
MRE11	Rabbit 1	4847	Cell Signaling Technology	1:1000 (IB)
ABCG2	Rabbit	42078	Cell Signaling Technology	1:1000 (IB)
Phospho-γH2A.X	Rabbit	9718	Cell Signaling Technology	1:200 (IF)
E-Cadherin (24E10)	Rabbit	3195	Cell Signaling Technology	1:1000 (IB); 1:200 (IF)
Galectin-3	Rabbit	87985	Cell Signaling Technology	1:1000 (IB); 1:200 (IF)
β-Actin	Mouse	3700	Cell Signaling Technology	1:4000 (IB)
Tubulin-Alexa 488	Mouse	8058	Cell Signaling Technology	1:100 (IF)
V5-Tag (D3H8Q)	Rabbit	13202	Cell Signaling Technology	1:200 (IF)
Vasa/DDX4	Mouse	MAB2030	R&D Systems	1:500 (IHC)
Polyclonal antibodies				
DDX4	Rabbit	Ab13840	Abcam	1:300 (IF)
UBE2L6	Rabbit	17278-1-AP	Proteintech	1:1000 (IB)
DNA2	Rabbit	18727-1-AP	Proteintech	1:1000 (IB)
DNM1	Rabbit	18205-1-AP	Proteintech	1:1000 (IB)
KIFAP3	Rabbit	12700-1-AP	Proteintech	1:1000 (IB)
RBBP6	Rabbit	11882-1-AP	Proteintech	1:1000 (IB)
FBXL14	Rabbit	13934-1-AP	Proteintech	1:1000 (IB)
Secondary antibodies				
Anti-rabbit Cy3	Goat	A10520	Thermo Fisher Scientific	1:300 (IF)
Anti-rabbit HRP	Goat	7074	Cell Signaling Technology	1:1000 (IB)
Anti-mouse HRP	Horse	7076	Cell Signaling Technology	1:1000 (IB)
Nuclear staining				
Hoechst 3342	N/A	H3579	Thermo Fisher Scientific	1:2000 (IF)

A list of chemicals used in this study.

**Table:** 

Drug name	Cat #	Vendor	Dose
Drugs used in CTG Assay			
Cisplatin	232120	Sigma-Aldrich	0.5–10 μM
Hu: Hydroxyurea	400046	Sigma-Aldrich	0.05–1 mM
CPT: Camptothecin	208925	Sigma-Aldrich	0.05–1 μM
ETP: Etoposide	341205	Sigma-Aldrich	1–20 μM
Inhibitors used in motility assay			
Ruxolitinib (JAK inhibitor)	S1378	Selleck Chemical	1 μM
Mirin (RAD50 and MRE inhibitor)	1198097970	Selleck Chemical	50 μM
LRRK2-IN-1 (DCLK1 inhibitor)	1234480842	Selleck Chemical	10 μM
Drugs used in other experiments			
Cisplatin (p-γH2AX staining and Cytokine profiling)	50-595-860	Fisher Scientific	10 μM
OPP (protein synthesis detection)	C10457	Thermo Fisher Scientific	1:1000

### Contact for reagent and resource sharing

For further information, requests should be directed to and will be fulfilled by the Lead Contact, Mamiko Yajima (Mamiko_Yajima@brown.edu).

### Experimental model and subject details

All human cancer cell lines were obtained from ATCC. Mouse studies were performed in accordance with the guidelines of the Institutional Animal Care and Use Committee at Kanazawa University. The procedures of cell injection and analysis of tumor growth were performed using KSN/Slc mice (Japan SLC, Inc.)^43^.

### Methods details

#### Cell lines, cell culture, and cell number counting

SCLC cell lines H69AR (ATCC# CRL-11351) and SHP77 (ATCC# CRL-2195), H69 (ATCC# HTP-119), H187 (ATCC# CRL-5804) and H82 (ATCC# HTB-175), as well as Non-SCLC (NSCLC) cell lines H1792 (ATCC# CRL-5895) and H23 (ATCC# CRL-5800) were cultured in RPMI-1640 (#A1049-01, GIBCO) supplemented with 10% (v/v) heat-inactivated fetal bovine serum (Thermo Fisher Scientific), and antibiotics, in a humidified atmosphere of 95% air and 5% CO_2_ at 37 °C. For all experiments, cells in the log phase of growth were used. For counting cell numbers, each cell line was suspended at 1 × 10^5^/mL and counted on a hemocytometer every 3 days of culture for five independent times.

#### General handling of cell lines

The original stocks of cells were stored as backups upon their arrival from ATCC. The morphology and behavior of the cells were carefully monitored during multiple rounds of passage. When an unexpected mutational phenotype appears, those cells will be discarded and replaced with the cells in the original stocks. For handling Knockout (KO) and Overexpression (OE) cell lines, upon lentiviral infection and antibiotics selection, original stocks of each line were stored in 5–10 separate vials, and only one each of the vials was used for the cycle of experiments. The level of DDX4-KO and OE was originally scored by multiple analyses, including immunoblot and immunofluorescence for both KO and OE cell lines, and further genomic PCR followed by sequencing to detect CRISPR/Cas9-mediated genomic mutations in KO cell lines as shown in Supplementary Figs. S1–4. The level of KO/OE in the original stocks was then used as a standard to monitor the level of KO/OE in passaging cells over time. Protein level was monitored by immunoblot analysis for each cycle of the experiments, and additional approaches such as PCR were also periodically used to determine the level of KO/OE. In general, these cells were used within three to six months of initial passage. Especially, for the KO cell lines, CRISPR/Cas9 genome editing impacts only transcription but not the existing mRNA or protein, therefore, many KO cells initially survived the antibiotics selection steps and passaging yet became more fragile over time. Therefore, we did not maintain the KO cell lines but rather used the fresh aliquots of the original cells or made fresh KO cell lines as much as possible for each set of experiments. Further, when the level of KO/OE was markedly compromised, these cells were discarded and replaced with the cells in the original stocks. For bulk KO/OE cell lines, once the original stocks are out, a new line of KO/OE cell lines was constructed again through lentiviral infection and antibiotics selection. All cell lines were also routinely screened for mycoplasma contamination.

#### Fluorescence-activated cell sorting (FACS) for five-cell selection

A five-colony selection of DDX4-knockout (KO) cell line was performed by FACS sorting (FACS Aria II, BD Biosciences). Five cells of each of the isolated cells were cultured in each well of the 96- or 24-well plate for ~2 months and subjected to genomic PCR and sequencing to identify the efficiency of KO.

#### DDX4 overexpression in H69AR and SHP77 cells

Lentiviral vectors for DDX4, DDX4-C3, and NanoLuc (control) overexpression were constructed by inserting DDX4 open reading frame (ORF), DDX4-C3 or NanoLuc sequence into the plx307 or plx304 vector that contains EF1-α promoter at its 5’ end using the Gateway cloning protocol (Thermo Fisher Scientific). DDX4-C3 and C3m1~m4 mutants were constructed by PCR-amplifying the DDX4-ORF fragment using a primer set containing the mutations to convert all or one of the last three amino acids of E-S-W-D to Alanine (see the resource table for details). Lentiviral infection was performed, followed by a puromycin and/or blasticidin selection at the concentration of 2–10 μg/mL^44,45^.

#### DDX4 CRISPR-mediated knockout in H69AR and SHP77 cells

Lentiviral vectors for CRISPR-mediated DDX4 knockout were previously constructed and used in myeloma IM9 cells, which resulted in a successful knockout of DDX4^28^. Briefly, three guide RNAs (gRNAs) were designed within the 3rd exon of the DDX4 gene locus (see also the resources table)^45,46^. A scrambled gRNA sequence formed by a random combination of A, G, T, and C, which does not share identity with either the mouse or human genome, was used as a control^44^. The efficiency of genome editing in each cell line was analyzed by genomic PCR and sequencing (Supplementary Fig. S2b), confirming mutations in the 3rd exon of the DDX4 genomic locus in 80% of H69AR-Sg1 cell lines, 10% of Sg2, and 60% of Sg3 cell lines (*n* = 10 each) (Supplementary Fig. S2c). In SHP77, those mutations were less efficient and 42% in Sg1 and 46% in Sg3 (*n* = 10 each) (Supplementary Fig. S3A). In both cell lines, no mutation was ever found in control SC cell lines (*n* = 11 each). Immunoblot and immunofluorescent results confirmed that these genomic mutations reduced DDX4 protein expression in the Sg1 and Sg3 lines compared to the control cell line SC (Supplementary Figs. S2d, e, S3b, c).

#### Genomic extraction and genomic PCR

In total, 1 μl of each cell pellet was subjected to genomic DNA extraction by treating with 20 μL of QuickExtract DNA Extraction Solution (# QE09050, Epicenter, USA) at 65 °C for 6 min followed by heat inactivation at 98 °C for 2 min. In all, 1 μl each of these treated samples was used for genomic PCR to amplify a flanking region of the 3rd exon of DDX4, where the gRNA sequences were designed, using high fidelity HIFI PCR premix (Clonetech, USA) with primers summarized in the resource table. The resultant PCR products were either separated by agarose gel electrophoresis and visualized or subjected to sub-cloning into the Zero Blunt TOPO PCR Cloning Vector (Invitrogen, USA) for sequencing.

#### Reverse transcription and quantitative real-time PCR (RT-qPCR)

RNA extraction was performed using RNeasy Mini Kit (QIAGEN, Hilden, Germany), and 1 μg each of the resultant total RNAs were subjected to Reverse Transcription (RT) using the Maxima First-Strand cDNA Synthesis Kit (Thermo Fischer Scientific, Waltham, MA) by following the manufacturer’s protocol. Overall, 1 μl each of the cDNAs was then used either for conventional PCR reactions or qPCR reactions. Conventional PCR reactions were performed with 35 cycles using HIFI PCR premix (Clonetech, USA). qPCR was performed using 1 μL of cDNA with Luna Universal qPCR Master Mix (# M3003S, New England Biolabs, USA) following the manufacturer’s protocol. The primers are summarized in the resource table.

#### Immunofluorescence, OPP staining, and microscopy

Cells were fixed with 4% PFA at 4 °C overnight or 30 min at room temperature, washed with PBS three times, and stained with a primary antibody of interest (see the resource table for details) at 1:100–1:300 dilution at room temperature for 3–5 h. These samples were washed six times with PBS and stained with a secondary antibody against rabbit IgG (1:500, 1 mg/mL, Invitrogen) in PBS. They were then washed four times and a Hoechst nuclear stain (10 mg/mL, Promega) in PBS was applied at a 1:1500 dilution. For OPP staining, cells were incubated with O-propargyl-puromycin (OPP, Thermo Fisher, # C10457) at 1:1000 dilution prior to fixation and subjected to Click-iT reaction by following the manufacturer’s protocol. The resultant cells were imaged by confocal laser microscopy (Olympus FV3000 and Olympus Spinning disk). Imaging was conducted using the same laser condition throughout each cycle of the experiments with both the control and experimental groups for quantitative accuracy. The signal intensity of each image was measured by *Image J* for quantitative analysis.

#### Immunoblot analysis

Immunoblots were performed by collecting ~10 μL of packed cells from each group in 50 μL of loading buffer. Each sample (2 μL) was run on a 10% Tris-glycine polyacrylamide gel and transferred to nitrocellulose membranes for immunoblotting with each primary antibody of interest at 1:1000 (see the resource table for details), and then with peroxidase-conjugated anti-mouse or -rabbit secondary antibodies at 1:2000 (Cell Signaling Technologies), respectively. The bound antibodies were detected by incubation in a chemiluminescence solution (1.25 mM luminol, 68 μM coumaric acid, 0.0093% hydrogen peroxide, and 0.1 M Tris pH 8.6) for 1–10 min, exposed to film and developed. Each experiment was performed at least three independent times. The signal intensity of each band was measured by *Image J* to construct graphs.

#### Cell motility assay

The cells (1 × 10^5^ cells/ml) from each group were cultured in each well of the 24-well plate and imaged every 30 min for H69AR cells or every minute for SHP77 cells in live up to desired time point. Images were taken with a 10X objective on an EVOS cell imaging microscope with a CO_2_ incubator (Thermo Fisher Scientific). For inhibitor treatment, each inhibitor at the desired dose (see the resources table for details) was added at the beginning of imaging for 24 h. Analyses were all done using *FIJI’s trackmate*. Cell motility was analyzed by measuring the position of the cell centroid at every time point and plotted to show the trace of centroid movement. The distance that the cell centroid traversed for each time point was calculated to determine the level of the movement.

#### Drug treatment and cell survivability (CTG) assay

For drug treatment, details are listed in the resource table or described in the text for each experiment. For the cell survivability assay, each sample group (1 × 10^5^ cells/ml) was harvested in each well of the 96-well plate, treated with a drug of interest or with DMSO (for control) with various concentrations for three days, and subjected to Cell Titer-Glo Luminescent Cell Viability (CTG) Assay (Promega, USA) by following the manufacturer’s protocol. The data was normalized by the value of the control (DMSO-treated) group.

#### Label-free quantitative proteomics and data analysis

For each cell line [LUC (Control) and DDX4-OE (Experimental)] 4 × 10^6^ H69AR cells were lysed with a lysis buffer (8 M urea, 1 mM sodium orthovanadate, 20 mM HEPES, 2.5 mM sodium pyrophosphate, 1 mM β-glycerophosphate, pH 8.0, 20 min, 4 °C) followed by sonication at 40% amplification by using a microtip sonicator (QSonica, LLC, Model no. Q55) and cleared by centrifugation (14,000×*g*, 15 min, 15 °C). Protein concentration was measured (Pierce BCA Protein Assay, Thermo Fisher Scientific, IL, USA) and a total of 100 µg of protein per sample was subjected to trypsin digestion. Tryptic peptides were desalted using C18 Sep-Pak plus cartridges (Waters, Milford, MA) and were lyophilized for 48 h to dryness. The dried eluted peptides were reconstituted in buffer A (0.1 M acetic acid) at a concentration of 1 µg/µl and 5 µl was injected for each analysis.

The LC-MS/MS was performed on a fully automated proteomic technology platform^47^ that includes an Agilent 1200 Series Quaternary HPLC system (Agilent Technologies, Santa Clara, CA) connected to a Q Exactive Plus mass spectrometer (Thermo Fisher Scientific, Waltham, MA). For the LC-MS/MS, the peptides were separated through a linear reversed-phase 90 min gradient from 0 to 40% buffer B (0.1 M acetic acids in acetonitrile) at a flow rate of 3 µl /min through a 3 µm 20 cm C18 column (OD/ID 360/75, Tip 8 µm, New objectives, Woburn, MA) for a total of 90 min run time^47^. The electrospray voltage of 2.0 kV was applied in a split-flow configuration, and spectra were collected using a top-9 data-dependent method. Survey full-scan MS spectra (*m/z* 400–1800) were acquired at a resolution of 70,000 with an AGC target value of 3 × 10^6^ ions or a maximum ion injection time of 200 ms. The peptide fragmentation was performed via higher-energy collision dissociation with the energy set at 28 normalized collision energy (NCE). The MS/MS spectra were acquired at a resolution of 17,500, with a targeted value of 2 × 104 ions or a maximum integration time of 200 ms. The ion selection abundance threshold was set at 8.0 × 10^2^ with charge state exclusion of unassigned and z = 1, or 6–8 ions, and a dynamic exclusion time of 30 s.

For database search and label-free quantitative analysis, peptide spectrum matching of MS/MS spectra of each file was searched against the human database (UniProt) using the Sequest algorithm within Proteome Discoverer v 2.3 software (Thermo Fisher Scientific, San Jose, CA). The Sequest database search was performed with the following parameters: trypsin enzyme cleavage specificity, two possible missed cleavages, 10 ppm mass tolerance for precursor ions, and 0.02 Da mass tolerance for fragment ions. Search parameters permitted variable modification of methionine oxidation (+15.9949 Da) and static modification of carbamidomethylation (+57.0215 Da) on cysteine. Peptide assignments from the database search were filtered down to a 1% FDR. The relative label-free quantitative and comparative among the samples were performed using the Minora algorithm and the adjoining bioinformatics tools of the Proteome Discoverer 2.3 software. To select proteins that show a statistically significant change in abundance between two groups, a threshold of 1.5-fold change with a p value (0.05) was selected. The data is deposited and available through jPOST (accession number: JPST001674) or ProteomeXchange (accession number: PXD034861).

#### Cytokine profiling

Multiplex assays were performed using Human Cytokine/Chemokine Magnetic Bead Panel (Cat. # HCYTMAG-60K-PX30) on a Luminex MAGPIX system (Merck Millipore)^44^. Briefly, conditioned media concentration levels (pg/mL) of each protein were determined by 5-parameter curve fitting models. The average of two replicate fold changes relative to the corresponding control was calculated and plotted as log^2^FC.

#### Animal studies

Mouse experiments were conducted in accordance with a Kanazawa University Institutional Animal Care and Use Committee–approved protocol. Female KSN/Slc nude mice (6–12 weeks) were subcutaneously injected on their armpits of right anterior limbs with 5 × 10^5^ cells of each group of H69AR cells and then monitored daily for tumor growth for 2–3 weeks until the tumor reaches 1500 mm^3^. Tumor volume was measured with a caliper and calculated by the formula, tumor size = *ab*^*2*^*/2*. *a* is the larger and *b* is the smaller of the two dimensions. When the xenografted tumors grew up to a mean tumor volume of around 250 mm^3^, tumor-bearing mice were intraperitoneally injected with various doses of Cisplatin (1 mg kg^−1^) or saline every other day for a total of six times^48^. Tumor volume and body weight were monitored over time. Tumor progression and regression were monitored daily. The tumor-bearing mice were sacrificed 39 days after treatment, and the xenografts, lung, and liver tissues were removed and subjected to RNA extraction for RT-qPCR or to 10% PFA fixation for Immunohistochemistry for hematoxylin and eosin staining.

RT-qPCR was as performed against the genes expressed in the vector such as human DDX4, NanoLuc, or Cas9 gene using primers or Taqman probe (Thermo Fisher Scientific) (primer sequences are summarized in the resources table). As controls, RNAs of the same tissue types derived from non-tumor-bearing mice were used. RNA extraction was performed using the RNeasy Mini Kit (Qiagen, Cat. # 74106). RNA samples (1 μg) were reverse-transcribed using Super- Script III First-Strand Synthesis SuperMix (Thermo Fisher Scientific, Cat. # 1683483). Quantitative real-time PCR was performed using Power SYBR Green PCR Master Mix (Thermo Fisher Scientific, Cat. # 4367659). Values represent the average of three technical replicates from at least two independent experiments (biological replicates).

#### Clinical data mining

From 79 tumor samples that were previously published^42^, fastq files were fetched from GEO (accession number GSE60052) and the clinical data was downloaded from the supplementary data provided in the paper. The other tumor data (ex. AML, ESCA, LUAD) were acquired from TCGA or TARGET by using an R package called TCGAbiolinks package. 48 SCLC patient samples had survival data which among them 40 samples were treatment naive. Non-tumor patient data were removed from all the tumor data. The samples were further divided into low and high DDX4 based on maximally ranked statistics. The survminer (version 0.4.6) and survival (3.1–8 version) R packages were used to perform survival analysis. EdgeR package in DEBrowser (version 1.2.4) was used to perform differential gene expression analysis at an adjusted *P* value cut-off of <0.01 and fold change >1.5. Counts were filtered in a way that the count distribution follows the normal distribution. The EnhancedVolcano R package was used to visualize the results of differentially expressed genes. Furthermore, these expressed genes were processed to gene sets enrichment and/or functional analysis on the following gene sets (inflammatory response and immune response, germline factors, and piwi RNA biogenesis). The results of enrichment and/or functional analysis were visualized by clusterProfiler.

### Statistics and reproducibility

All images were analyzed by Image J (NIH), and statistical significance was performed by PRISM (GraphPad) using one-way or two-way ANOVA followed by the Tukey post hoc test. *P* values less than 0.05 were considered significant. Asterisks were used to indicate significance corresponding with *P < 0.05, **P < 0.01, ***P < 0.001, ****P < 0.0001. Columns represent means ± SD or SEM. For reproducibility, each experiment was repeated at least twice. Most of the experiments were repeated 3–5 times, yet only the cycles of experiments with technical confidence were processed for analysis. All attempts at replication were successful. The sample size was determined by the condition that the analysis provides consistent trends across multiple experimental cycles with statistical significance. For technically or financially challenging experiments, three representative samples with technical confidence were processed for the analysis. To increase confidence in our results, multiple different experiments were conducted to address the same question, which was then combined to make a single conclusion. Lastly, this study was contributed by multiple authors using the same or similar cells, constructs, and technologies multiple times across the article, which resulted in the same or similar results, providing a natural randomization.

### Reporting summary

Further information on research design is available in the Nature Portfolio Reporting Summary linked to this article.

## Supplementary information

Supplementary Information

Description of Additional Supplementary Data

Supplementary Movie 1

Supplementary Movie 2

Supplementary Movie 3

Supplementary Movie 4

Reporting Summary-New

Description of Additional Supplementary Data

Supplementary Data 1

Supplementary Data 2

## Data Availability

The proteomics data is available in Supplementary Data 1 or through jPOST (accession number: JPST001674) and ProteomeXchange (accession number: PXD034861). Other datasets used and/or analyzed during the current study are available in Supplementary Data 2. The Blots data are available in Supplementary Fig. 10.

## Code availability

The codes and software used in this study are widely or publicly available. The codes are available from the corresponding author as needed.
