# Supplementary material for: The germline factor DDX4 contributes to the chemoresistance of small cell lung cancer cells
Source: Commun Biol. 2023 Jan 18;6:65. doi: 10.1038/s42003-023-04444-7 (PMC9849207; doi:10.1038/s42003-023-04444-7)
Supplement: Supplementary file 1 — Supplementary Information [file 42003_2023_4444_MOESM1_ESM.pdf]

SUPPLEMENTARY MATERIALS

Title: The germline factor DDX4 contributes to the chemoresistance of small cell lung cancer cells

Authors: Christopher Noyes[*1], Shunsuke Kitajima[*2,3], Fengkai Li[4], Yusuke Suita[5], Saradha Miriyala[5], Shakson Isaac[1], Nagib Ahsan[6,7], Erik Knelson[2], Amir Vajdi[8], Tetsuo Tani[2], Tran C. Thai[2], Derek Xu[1], Junko Murai[9], Nikos Tapinos[7], Chiaki Takahashi[4], David A. Barbie[2] and Mamiko Yajima[1]

This file contains:

1. **Supplementary Figures and Legends**
2. **Supplementary Movie legends**

SUPPLEMENTARY FIGURES & LEGENDS

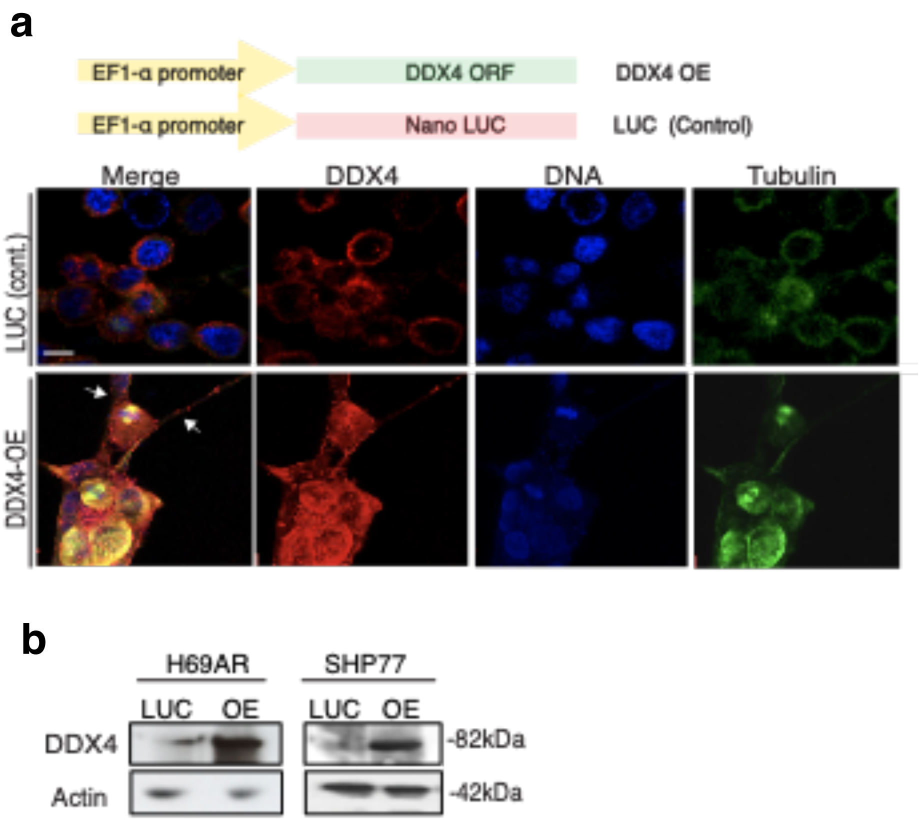

Fig. S1. DDX4 overexpression in H69AR and SHP77 cells. **(a)** DDX4-ORF was driven by the EF1-α promoter (upper diagram), which showed overexpression of DDX4 protein (red) by immunofluorescence in H69AR cells. DDX4-OE cell lines showed significantly different morphology characterized by flattened shape with extended filopodia (arrows). Tubulin, green; DNA, blue. Scale bar = 5 µm. **(b)** Increased DDX4 protein level (red) was confirmed by immunofluorescence.

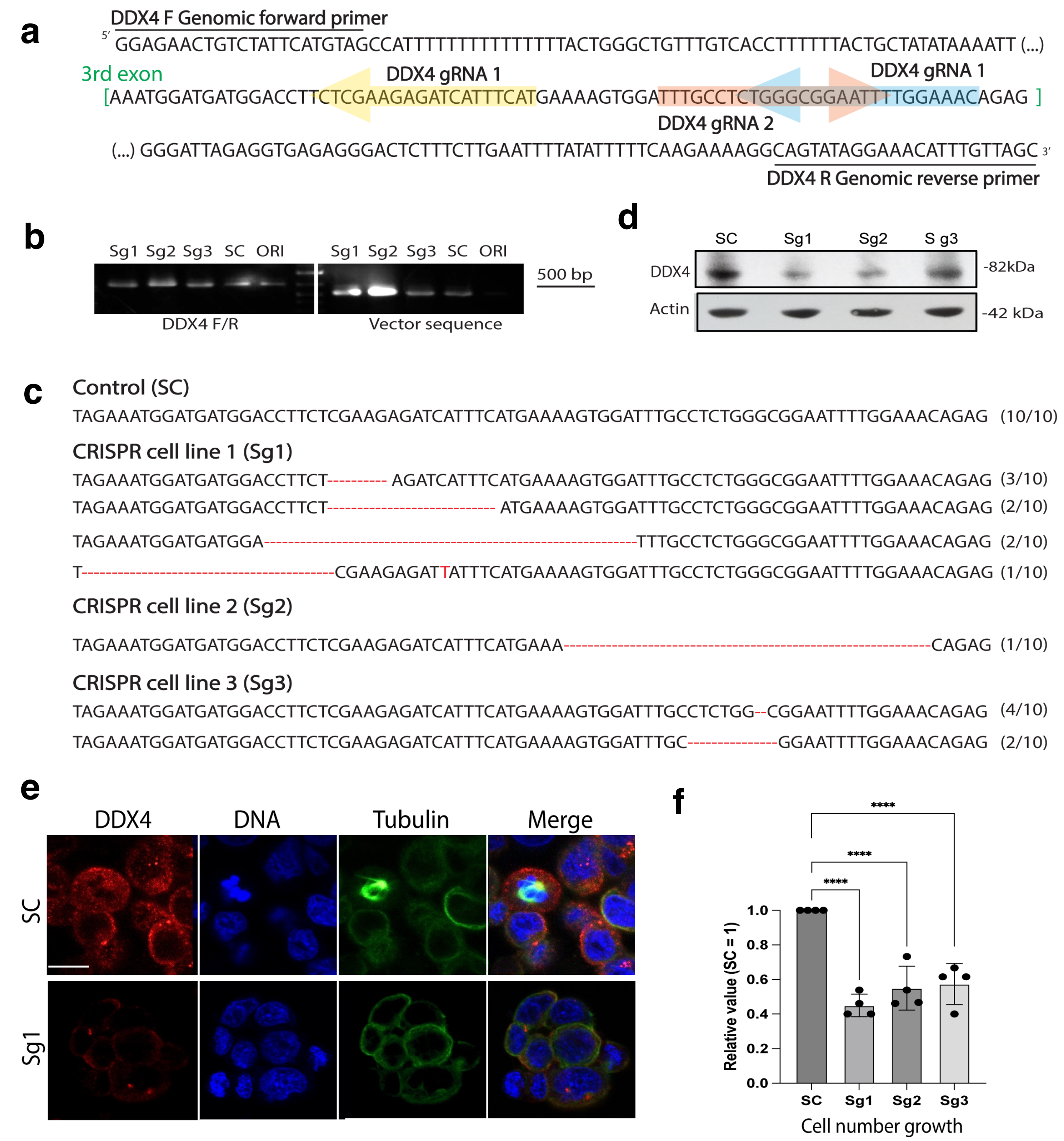

Fig. S2. DDX4 knockout cell construction in H69AR by CRISPR-Cas9 gene-editing technology. **(a)** Three different gRNAs were designed within the 3rd exon of the DDX4 gene to construct knockout cell lines (Sg1-Sg3). **(b)** Left, Genomic PCR results of the DDX4 genomic locus shown in A for three CRISPR knockout cell lines (Sg1-Sg3), control cells introduced with scrambled gRNA sequence (SC), and parental intact cells (ORI). Right, Genomic PCR results detect the lentiviral vector sequence used for virus infection, showing the effective introduction of CRISPR constructs in each cell line. **(c)** DNA sequencing results of the 3rd exon and its flanking region of the DDX4 gene are depicted in (a). The Sg1 cell line showed deletions in many of the clones sequenced compared to other KO lines, and the control (SC) line showed no mutations. () in the right corner suggests the number of the genomic PCR clones showing the corresponding sequence. **(d-e)** Immunoblot (d) and Immunofluorescence (e) results showed reduced DDX4 protein expression in Sg1 and Sg2 knockout cell lines compared to control (SC). Actin was used as a loading standard for Immunoblot. **(f)** The cell number growth comparison among DDX4-KO cell lines. The same number ($1x10^5$) of cells was harvested for each cell line and the total number of cells was counted on Day 4 before passaging. Each value was normalized to that of the control line (SC). The results are the average of four independent experiments. One-way ANOVA was used for the graph. **** is $p < 0.0001$.

a

CRISPR cell line 1 (Sg1) (58. 3%)

TAGAAATGGATGATGGACCTTCTCGAAGAGATCATTTCATGAAAAGTGGATTTGCCTCTGGGCGGAATTTTGGAAACAGAG (5/12)

TAGAAATGGATGATGGACCTTCTCGAAGAGATCATTTCATGAAAAGTGGAT--------GGG---AATTTTGGAAACAGAG (1/12)

TAGAAATGGATGATGGACCTTCTCGAAGAGATCATTTCATGAAAAGTGGATTTGC--------GGAATTTTGGAAACAGAG (1/12)

TAGAAATGGATGATGGACCTTCTCGAAGAGATCATTTCATGAAAAGTGGATTTGCCTCTGGGGCGGAATTTTGGAAACAGAG (1/12)

TAGAAATGGATGATGGACCTTCTCGAAGAGATCATTTCATGAAAAGTGGATTTGCCTCTGG-CGGAATTTTGGAAACAGAG (1/12)

TAGAAATGGATGATGGACCTTCTCGAAGAGATCATTTCATGAAAAGTGGATTTGCCTCTGG------------------ (1/12)

TAGAAATGGATGATGGACCTTCTCGAAGAGATCATTTCA-- (1/12)

TAGAAATGGATGATGGACCTTCTCGAAGAGATCATTTCATGAAAAGTGGTAAGCATCTTTGTCTTTCCTTAATCTCCTGAAC
TGTTGAATAATTGGATATATAGAGGCTTTCATGGCCATTTGGTACAAAGCTAAGTCTGTTGTAAGTCAGTGTGTAGTAGACTT
GGTGTTGAGATAATGAGTTCATCATTTTTCCAAGTTC------------------------------ (1/12)

CRISPR cell line 3 (Sg3) (54.5%)

TAGAAATGGATGATGGACCTTCTCGAAGAGATCATTTCATGAAAAGTGGATTTGCCTCTGGGCGGAATTTTGGAAACAGAG (5/11)

----------------------------GATCATTTCATGAAAAGTGGATTTGCCTCTGGGCGGAATTTTGGAAACAGAG (1/11)

--------------------------GAGATCATTTCATGAAAAGTGGATTTGCCTCTGGGCGGAATTTTGGAAACAGAG (1/11)

----------------------------GATCATTTCATGAAAAGTGGATTTGTGAAAAGTGGATTTGCCTCTGGGCGGAATTTTGGAAACAGAG (1/11)

TAGAAATGGATGAT-----------------------------------TTGCCTCTGGGCGGAATTTTGGAAACAGAG (1/11)

TAGAAATGGATGATGGACCTTC-----------ATTTCATGAAAAGTGGATTTGCCTCTGGGCGGAATTTTGGAAACAGAG (1/11)

--GATTTGCCTCTGGGCGGAATTTTGGAAACAGAG (1/11)

b

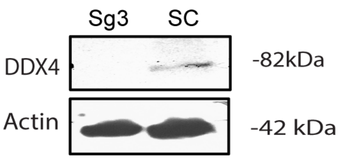

c

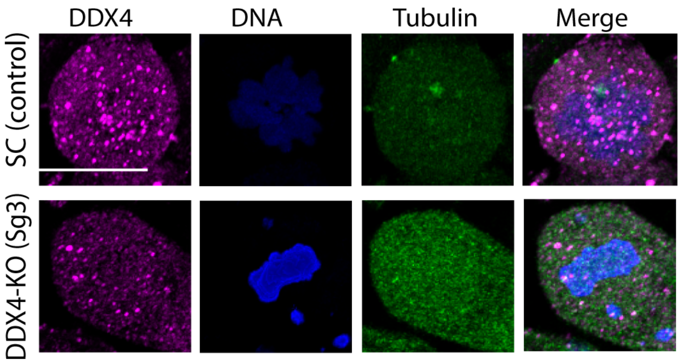

Fig. S3. DDX4 knockout cell construction in SHP77. **(a)** DDX4-KO was constructed by CRISPR-Cas9 gene-editing technology. The same lentivirus (Sg1 & Sg2) used in Fig. S2 for H69AR cells was used in SHP77 cells. The Sg3 cell line showed deletions in many of the clones sequenced compared to other KO lines in the 3rd exon of the DDX4 gene. The control (SC) line showed no mutations. () in the right corner suggests the number of the genomic PCR clones showing the corresponding sequence. **(b-c)** Immunoblot (b) and immunofluorescence (c) results showed reduced DDX4 protein expression (magenta) in Sg3 knockout cell lines compared to control (SC). Tubulin, green; DNA, blue. Scale bar = 5 μm.

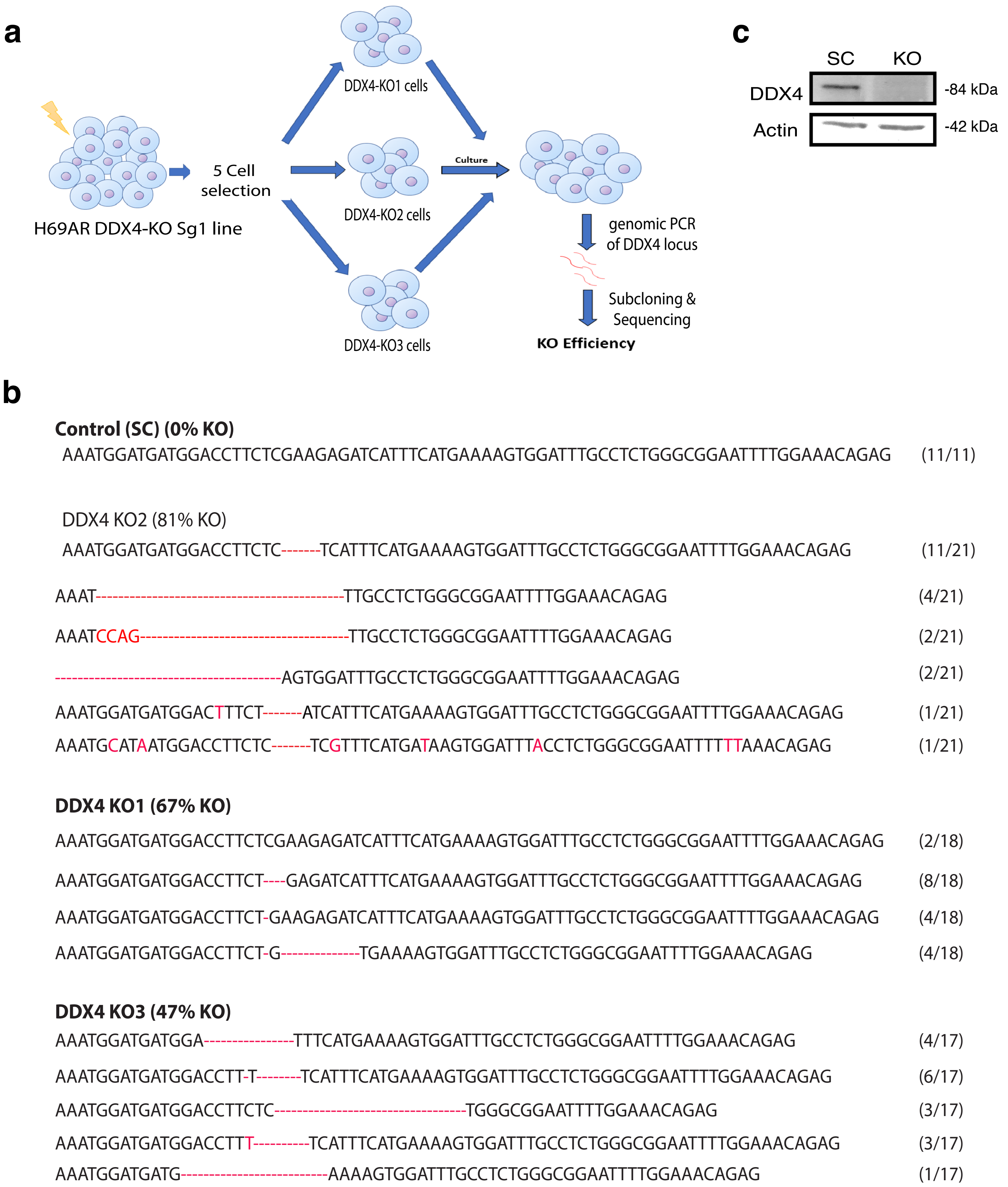

Fig. S4. Five-cell selection of the DDX4-KO lines in H69AR cells.
(a) A schema showing the process for the five-cell selection of DDX4-KO cells. The five groups were selected for DDX4-KO Sg1 and SC lines, respectively. Since only three out of five groups of DDX4-KO survived, the genomic PCR analysis was performed for these three groups (KO1, KO2, KO3). **(b)** KO2 exhibited the greatest DDX4-KO efficiency by inducing a stop codon either within or immediately after the 3rd exon of DDX4, whereas the same five-cell selection of SC lines resulted in no mutation. () in the right corner suggests the number of the genomic PCR clones showing the corresponding sequence. **(c)** Immunoblot results of SC (control) and KO (DDX4-KO) cells. Actin was used as a loading standard.

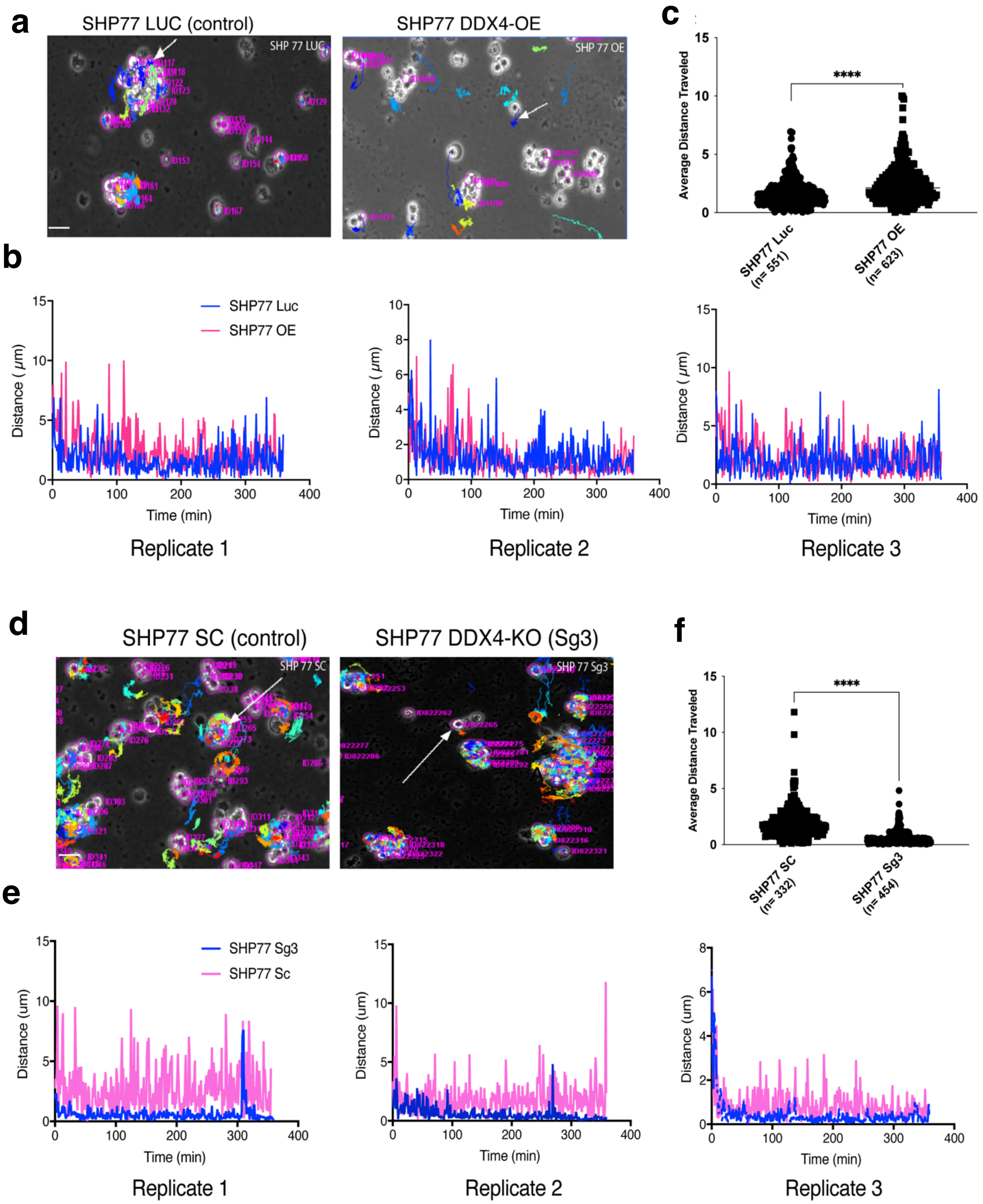

Fig. S5. DDX4 increased cell motility in SHP77 cells.

Cell lines of DDX4-OE and LUC (a-c) or of DDX4-KO and SC (d-f) were timelapse-imaged for 6~12 hours, with a new image being taken every minute. **(a & d)** The representative images of the paths (colored lines) taken by the tracked cells are shown. **(b & e)** The travel distances of the cells pointed to by the arrows were compared among cell lines and shown for each cell over the time point. **(c & f)** Travel distances per 30 minutes of all cells in the field were determined by summating and averaging the distance value at each time point for each cell in the field. () indicates the total number of cells analyzed. DDX4-KO (Sg3) showed lower average motility and DDX4-OE showed higher when compared to controls. Scale bar = 10 µm. An unpaired t-test was used for all graphs. **** is $p < 0.0001$.

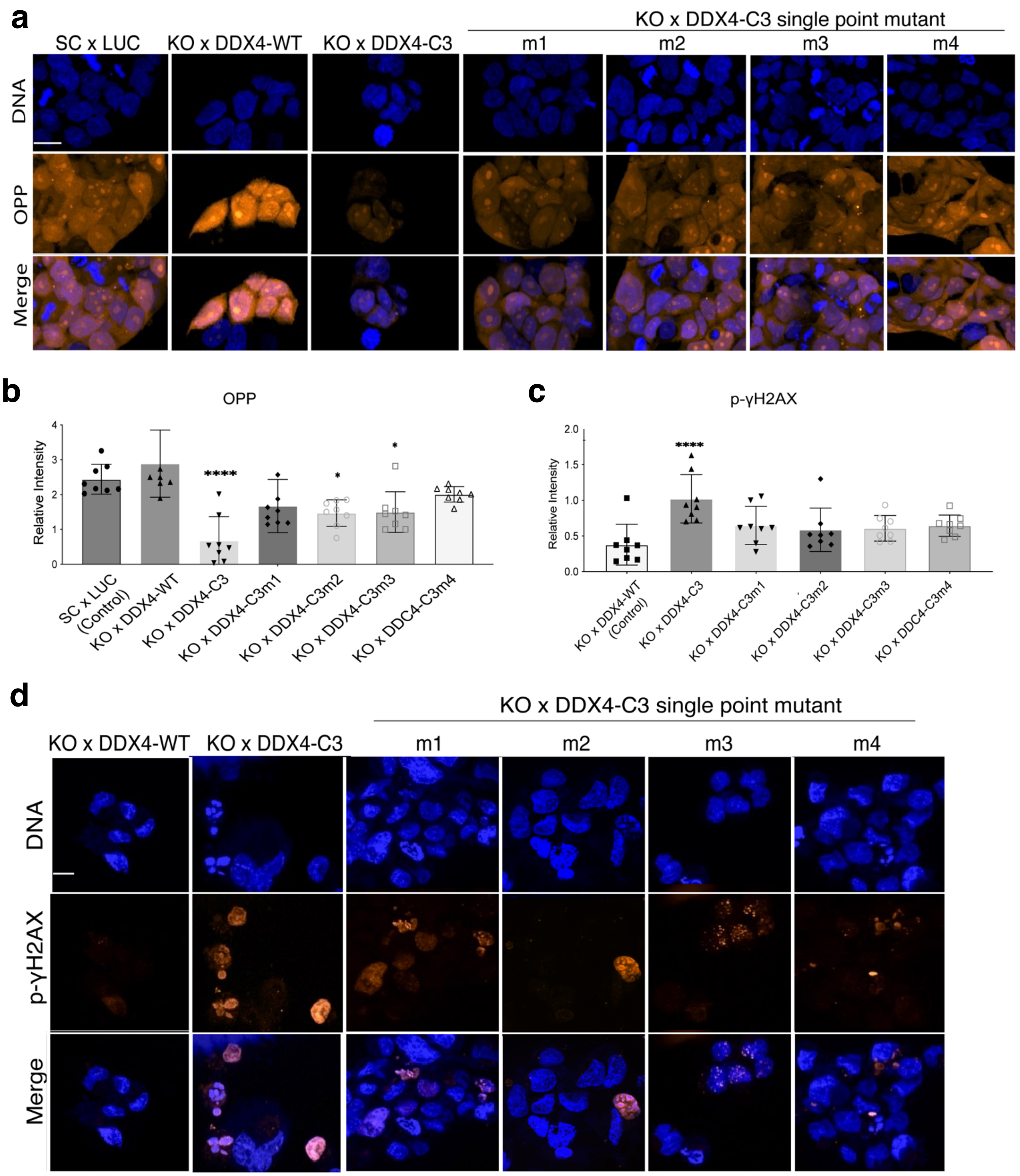

Fig. S6. A single-point mutation in the C3 region of DDX4 rescued the DDX4-KO phenotypes in H69AR cells. Translational activity **(a-b)** or DNA damage level **(c-d)** was measured by the OPP signal or by the p-γH2AX signal level for each sample group, respectively. The OPP or p-γH2AX signal intensity was normalized to the DNA signal level in the same ROI for each image. Graphs b and c, 8 ROIs were measured per sample group and the average value of each group is shown in the graph; n=3 biologically independent experiments. Scale bars = 10 µm. One-way ANOVA was used for all graphs. * is $p < 0.05$; **** is $p < 0.0001$. Columns represent means ± SD or SEM. All scale bars =5 µm.

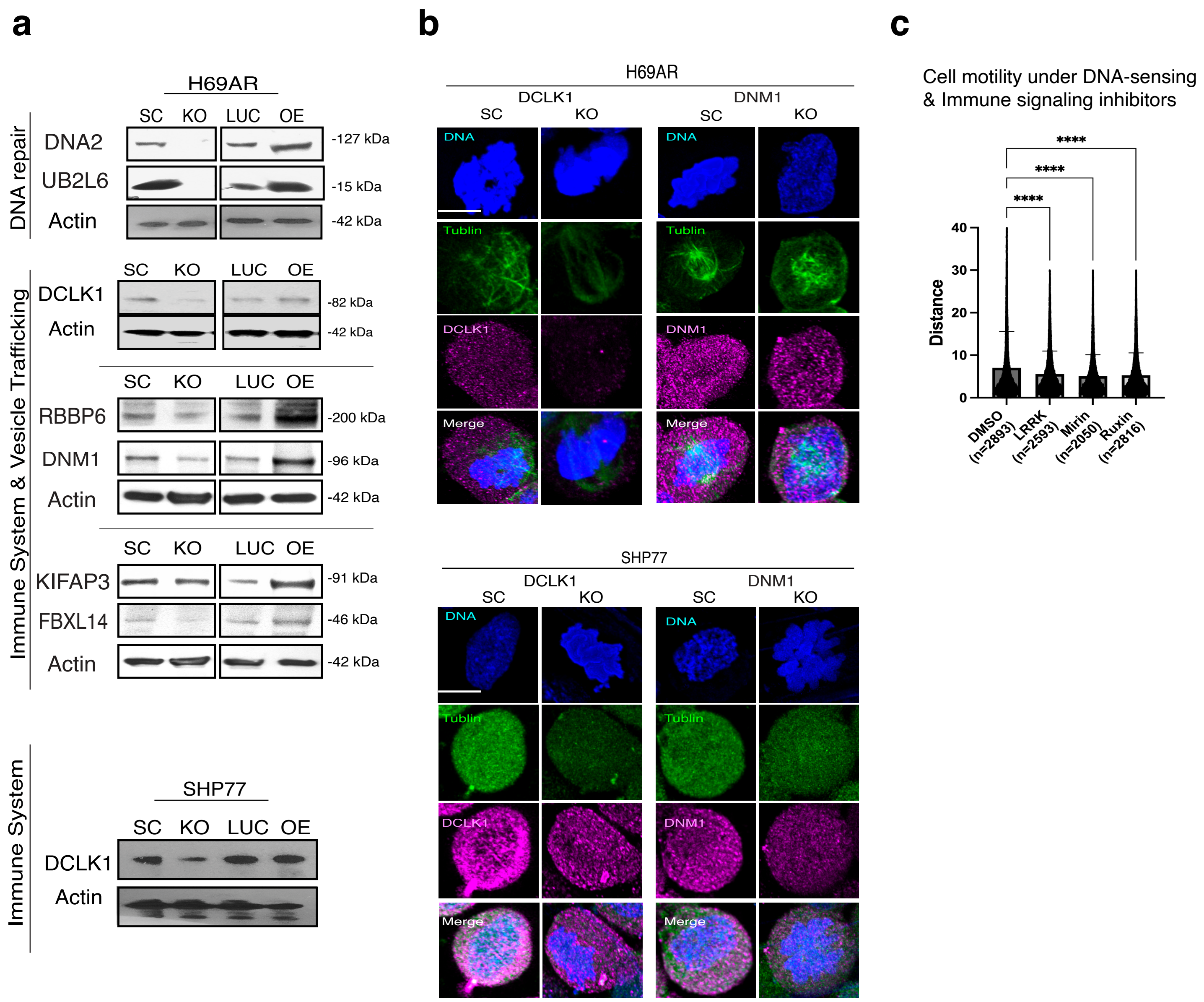

Fig. S7. DDX4 expression upregulated the metabolic pathways in H69AR and SHP77 cells.
(a-b) Validation of the proteomic results by immunoblot (a) or by immunofluorescence (b). DNA2, UB2L6, DCLK1, RBBP6, DNM1, KIFAP3, and FBXL14 levels were upregulated or downregulated by DDX4-OE or –KO, respectively, of H69AR or SHP77 cells. Actin was used as a standard for all immunoblots. Scale bars = 5 μm. DNA, blue; Tubulin, green. **(c)** Inhibitors for immune response (LRRK and Ruxin) and DNA-damage sensing (Mirin) all reduced cell motility in DDX4-OE cells. An unpaired t-test was used for the graph in C. **** is $p < 0.0001$. Columns represent means ± SD or SEM.

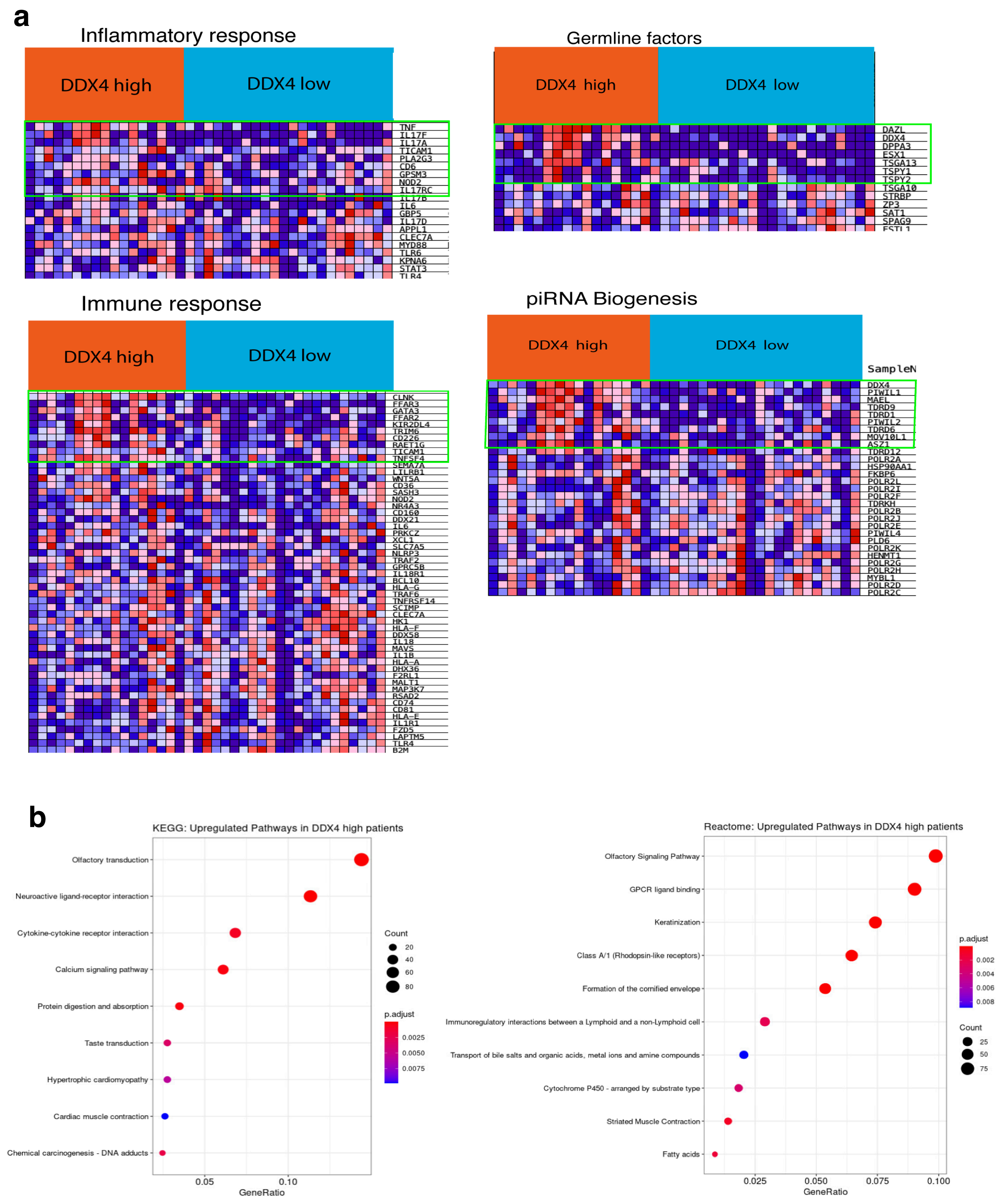

Fig. S8. Heat-maps (a) and functional analysis (b) for enriched genes of each category in the DDXD4-High SCLC patients.

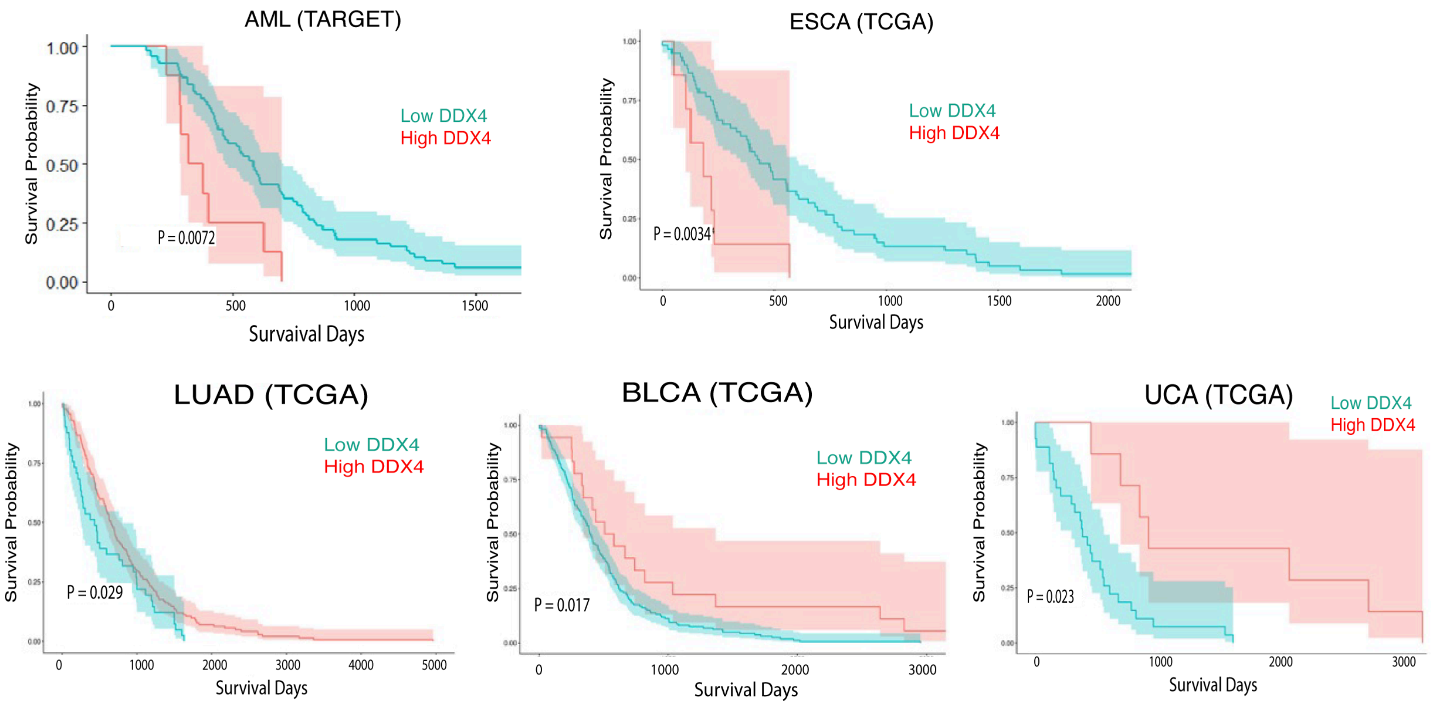

Fig. S9. Survival analyses of DDX4-high and -low patients, linked to Fig. 6e. mRNA expression dataset of AML patients' samples were obtained from TARGET and those of ESCA, LUAD, BLCA, and UCA patients' samples were all obtained from TCGA.

SUPPLEMENTARY MATERIALS

Title: The germline factor DDX4 contributes to the chemoresistance of small cell lung cancer cells

Authors: Christopher Noyes[*1], Shunsuke Kitajima[*2,3], Fengkai Li[4], Yusuke Suita[5], Saradha Miriyala[5], Shakson Isaac[1], Nagib Ahsan[6,7], Erik Knelson[2], Amir Vajdi[8], Tetsuo Tani[2], Tran C. Thai[2], Derek Xu[1], Junko Murai[9], Nikos Tapinos[7], Chiaki Takahashi[4], David A. Barbie[2] and Mamiko Yajima[1]

This file contains:

1. **Supplementary Figures and Legends**
2. **Supplementary Movie legends**

Immunoblots

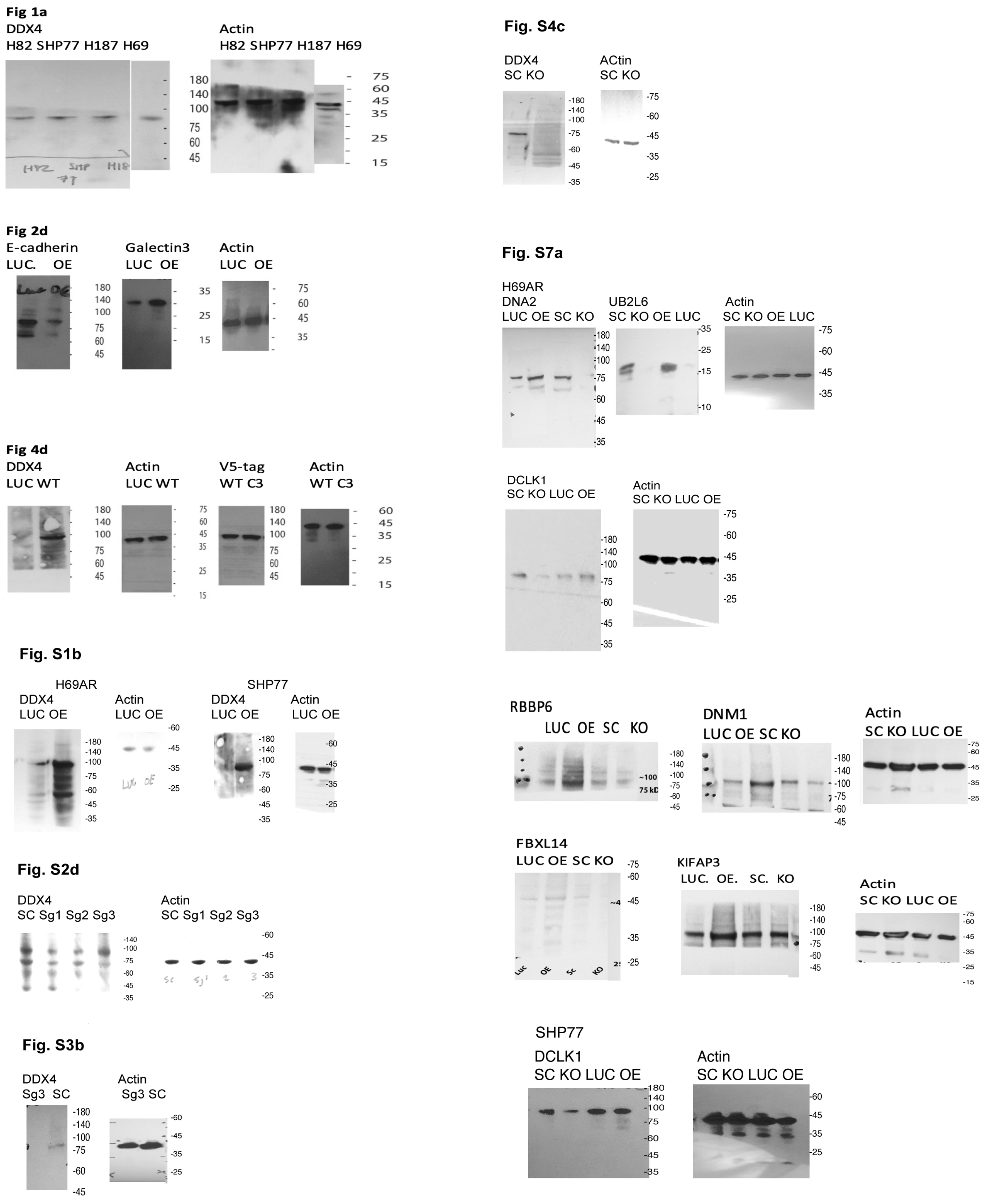

Fig. S10. The compilation of Immunoblots.

SUPPLEMENTARY MOVIE LEGENDS

M1-M4. Each LUC (M1), DDX4-OE (M2), SC(M3), and DDX4-KO (M4) H69A cell line was time-lapse imaged at 30 minutes intervals for 24~48 hours.
