# Supplementary material for: The germline factor DDX4 contributes to the chemoresistance of small cell lung cancer cells
Source: Commun Biol. 2023 Jan 18;6:65. doi: 10.1038/s42003-023-04444-7 (PMC9849207; doi:10.1038/s42003-023-04444-7)
Supplement: Supplementary file 2 — Description of Additional Supplementary Data [file 42003_2023_4444_MOESM2_ESM.docx]

**Description of Additional Supplementary Files**

**File name:**

**Description:**
