# Supplementary material for: The germline factor DDX4 contributes to the chemoresistance of small cell lung cancer cells
Source: Commun Biol. 2023 Jan 18;6:65. doi: 10.1038/s42003-023-04444-7 (PMC9849207; doi:10.1038/s42003-023-04444-7)
Supplement: Supplementary file 7 — Reporting Summary-New [file 42003_2023_4444_MOESM7_ESM.pdf]

nature portfolio

Corresponding author(s): Mamiko Yajima

Last updated by author(s): Dec 28, 2022

Reporting Summary

Nature Portfolio wishes to improve the reproducibility of the work that we publish. This form provides structure for consistency and transparency in reporting. For further information on Nature Portfolio policies, see our Editorial Policies and the Editorial Policy Checklist.

Statistics

For all statistical analyses, confirm that the following items are present in the figure legend, table legend, main text, or Methods section.

n/a	Confirmed	
☐	☒	The exact sample size (*n*) for each experimental group/condition, given as a discrete number and unit of measurement
☐	☒	A statement on whether measurements were taken from distinct samples or whether the same sample was measured repeatedly
☐	☒	The statistical test(s) used AND whether they are one- or two-sided<br>*Only common tests should be described solely by name; describe more complex techniques in the Methods section.*
☐	☒	A description of all covariates tested
☐	☒	A description of any assumptions or corrections, such as tests of normality and adjustment for multiple comparisons
☐	☒	A full description of the statistical parameters including central tendency (e.g. means) or other basic estimates (e.g. regression coefficient) AND variation (e.g. standard deviation) or associated estimates of uncertainty (e.g. confidence intervals)
☐	☒	For null hypothesis testing, the test statistic (e.g. *F*, *t*, *r*) with confidence intervals, effect sizes, degrees of freedom and *P* value noted<br>*Give P values as exact values whenever suitable.*
☒	☐	For Bayesian analysis, information on the choice of priors and Markov chain Monte Carlo settings
☒	☐	For hierarchical and complex designs, identification of the appropriate level for tests and full reporting of outcomes
☒	☐	Estimates of effect sizes (e.g. Cohen's *d*, Pearson's *r*), indicating how they were calculated

Our web collection on statistics for biologists contains articles on many of the points above.

Software and code

Policy information about availability of computer code

Data collection	Microscope image data was collected directly from the associated software (Olympus Fluoview (V4.2); EVOS M5000) attached to each microscope as indicated in the methods section.
Data analysis	Image J (ver. 1.53a), PRISM (ver 8), EXCEL (ver. 16.59)

For manuscripts utilizing custom algorithms or software that are central to the research but not yet described in published literature, software must be made available to editors and reviewers. We strongly encourage code deposition in a community repository (e.g. GitHub). See the Nature Portfolio guidelines for submitting code & software for further information.

Data

Policy information about availability of data

All manuscripts must include a data availability statement. This statement should provide the following information, where applicable:

- Accession codes, unique identifiers, or web links for publicly available datasets
- A description of any restrictions on data availability
- For clinical datasets or third party data, please ensure that the statement adheres to our policy

The datasets generated during and/or analysed during the current study are available from the corresponding author on reasonable request.

Field-specific reporting

Please select the one below that is the best fit for your research. If you are not sure, read the appropriate sections before making your selection.

☒ Life sciences ☐ Behavioural & social sciences ☐ Ecological, evolutionary & environmental sciences

For a reference copy of the document with all sections, see nature.com/documents/nr-reporting-summary-flat.pdf

Life sciences study design

All studies must disclose on these points even when the disclosure is negative.

Sample size

The sample size was determined by the condition that the analysis provides consistent trends across multiple experimental cycles with statistical significance. For technically or financially challenging experiments, three representative samples with technical confidence were processed for the analysis. To increase the confidence in our results, multiple different experiments were conducted to address the same question, which was then combined to make a single conclusion.

Data exclusions

The batches where the control groups showing significant defects (e.g. contamination, deformed morphology) were excluded from the analysis.

Replication

Each experiment was repeated at least twice. Most of experiments were repeated 3-5 times, yet only the cycles of experiments with technical confidence were processed for analysis. All attempts at replication were successful.

Randomization

This article was contributed by multiple authors using the same or similar cells, constructs and technologies multiple times across the article, which resulted in the same or similar results, providing a natural randomization.

Blinding

Cell and animal handling and advanced live imaging requires a highly trained skill and eyes to confirm no technical mistake is involved in each experiment. Blinding was therefore not appropriate in this article.

Reporting for specific materials, systems and methods

We require information from authors about some types of materials, experimental systems and methods used in many studies. Here, indicate whether each material, system or method listed is relevant to your study. If you are not sure if a list item applies to your research, read the appropriate section before selecting a response.

Materials & experimental systems

n/a	Involved in the study	
☐	☒	Antibodies
☐	☒	Eukaryotic cell lines
☒	☐	Palaeontology and archaeology
☐	☒	Animals and other organisms
☒	☐	Human research participants
☒	☐	Clinical data
☒	☐	Dual use research of concern

Methods

n/a	Involved in the study	
☒	☐	ChIP-seq
☒	☐	Flow cytometry
☒	☐	MRI-based neuroimaging

Antibodies

Antibodies used

mAbs: DDX4, Cortactin, RAD50, AGCG2, DCLK1, MRE11, ABCG2, p-gamma-H2AX, E-Cadherin, Galetin-3, Beta-Actin, Tubulin-Alexa488, V5-Tag
pAbs: DDX4, UBE2L6, DNA2, DNM1, KIFAP3, RBBP6, FBXL14
2ndary Abs: Cy3-Rabbit IgG, HRP-rabbit IgG, HRP-mouse IgG.
Details are summarized in the Table in the Methods section.

Validation

Validations were performed by the manufacturer as below:
DDX4: https://www.cellsignal.com/products/primary-antibodies/ddx4-d10c5-rabbit-mab/8761
Cortactin: https://www.cellsignal.com/products/primary-antibodies/cortactin-h222-antibody/3503
RAD50: https://www.cellsignal.com/products/primary-antibodies/rad50-antibody/3427
AGCG2: https://www.cellsignal.com/products/primary-antibodies/abcg2-d5v2k-xp-rabbit-mab/42078
DCLK1: https://www.cellsignal.com/products/primary-antibodies/dclk1-dcamkl1-d2u3l-xp-rabbit-mab/62257
MRE11: https://www.cellsignal.com/products/primary-antibodies/mre11-31h4-rabbit-mab/4847
ABCG2: https://www.cellsignal.com/products/primary-antibodies/abcg2-d5v2k-xp-rabbit-mab/42078
pgamma-H2AX: https://www.cellsignal.com/products/primary-antibodies/phospho-histone-h2a-x-ser139-20e3-rabbit-mab/9718
E-Cadherin: https://www.cellsignal.com/products/primary-antibodies/e-cadherin-24e10-rabbit-mab/3195
Galectin-3: https://www.cellsignal.com/products/primary-antibodies/galectin-3-lgals3-d4i2r-xp-rabbit-mab/87985

The below Antibodies are widely used across organisms and applications. Validations were performed by the manufacturer as below:
Beta-Actin: https://www.cellsignal.com/products/primary-antibodies/b-actin-8h10d10-mouse-mab/3700
Tublin-Alexa488: https://www.cellsignal.com/products/antibody-conjugates/a-tubulin-dm1a-mouse-mab-alexa-fluor-488-conjugate/8058
V5-Taga; https://www.cellsignal.com/products/primary-antibodies/v5-tag-d3h8q-rabbit-mab/13202

Eukaryotic cell lines

Policy information about cell lines

Cell line source(s)	All cell lines were obtained from ATCC
Authentication	ATCC comprehensively performs authentication and quality-control tests on all distribution lots of cell lines
Mycoplasma contamination	The original lines from were tested by PCR and aliquoted. Each vial of cells was then used up within 10 passages.
Commonly misidentified lines (See ICLAC register)	N/A

Animals and other organisms

Policy information about studies involving animals; ARRIVE guidelines recommended for reporting animal research

Laboratory animals	Female KSN/Slc, Nude Mice (Mus musculus), 6-12 weeks of age.
Wild animals	No wild animals were used in the study.
Field-collected samples	No field-collected animals were used in the study.
Ethics oversight	Followed the Kanazawa University Institutional Animal Care and Committee–approved protocol.

Note that full information on the approval of the study protocol must also be provided in the manuscript.
