# Supplementary material for: The germline factor DDX4 contributes to the chemoresistance of small cell lung cancer cells
Source: Commun Biol. 2023 Jan 18;6:65. doi: 10.1038/s42003-023-04444-7 (PMC9849207; doi:10.1038/s42003-023-04444-7)
Supplement: Supplementary file 8 — Description of Additional Supplementary Data [file 42003_2023_4444_MOESM8_ESM.pdf]

Description of Additional Supplementary Files

File name: Supplementary Data 1
Description: Proteomics data.

File name: Supplementary Data 2
Description: Other datasets used and/or analyzed during the current study.
